# ABL1/2 and DDR1 Drive MEKi Resistance in NRAS-Mutant Melanomas by Stabilizing RAF/MYC/ETS1 and Promoting RAF Homodimerization

**DOI:** 10.3390/cancers15030954

**Published:** 2023-02-02

**Authors:** Anastasia Lyon, Rakshamani Tripathi, Christina Meeks, Daheng He, Yuanyuan Wu, Jinpeng Liu, Chi Wang, Jing Chen, Haining Zhu, Sujata Mukherjee, Saptadwipa Ganguly, Rina Plattner

**Affiliations:** 1 Department of Pharmacology and Nutritional Sciences, College of Medicine, University of Kentucky, Lexington, KY 40536, USA; 2 Biostatistics and Bioinformatics Shared Resource Facility, College of Medicine, Markey Cancer Center, University of Kentucky, Lexington, KY 40508, USA; 3 Department of Molecular and Cellular Biochemistry, University of Kentucky, Lexington, KY 40536, USA

**Keywords:** whole exome sequencing, RNA sequencing, melanoma, NRAS, DDR1, ABL1, ABL2, BRAF, CRAF, ARAF, ETS1, MYC, β-catenin, p27/KIP1

## Abstract

**Simple Summary:**

*NRAS*-mutant melanoma is a highly aggressive subtype with few treatment options. Although both *BRAF*-mutant and *NRAS*-mutant melanomas have activation of the MEK/ERK pathway, MEK inhibitors (MEKi) are only effective for the *BRAF*-mutant subtype. The aim of this study was to understand why MEKi are ineffective in *NRAS*-mutant melanomas with the long-term goal of identifying new treatment regimens. Here, we show that ABL and DDR kinases are critically important for MEKi resistance because they cooperate to promote the stability of key proteins involved in driving melanoma growth and survival. FDA-approved drugs that inhibit ABL1/2 and DDR1 have been used for decades to treat leukemia. We showed that one such inhibitor prevents MEKi resistance from developing in a *NRAS*-mutant melanoma animal model. Thus, the data in this study provide the rationale for testing the use of drugs targeting ABL1/2 and DDR1 in combination with MEKi for patients with *NRAS*-mutant melanomas who have failed to respond to immunotherapy.

**Abstract:**

Melanomas harboring *NRAS* mutations are a particularly aggressive and deadly subtype. If patients cannot tolerate or the melanomas are insensitive to immune checkpoint blockade, there are no effective 2nd-line treatment options. Drugs targeting the RAF/MEK/ERK pathway, which are used for *BRAF*-mutant melanomas, do little to increase progression-free survival (PFS). Here, using both loss-of-function and gain-of-function approaches, we show that ABL1/2 and DDR1 are critical nodes during *NRAS*-mutant melanoma intrinsic and acquired MEK inhibitor (MEKi) resistance. In some acquired resistance cells, ABL1/2 and DDR1 cooperate to stabilize RAF proteins, activate ERK cytoplasmic and nuclear signaling, repress p27/KIP1 expression, and drive RAF homodimerization. In contrast, other acquired resistance cells depend solely on ABL1/2 for their survival, and are sensitive to highly specific allosteric ABL1/2 inhibitors, which prevent β-catenin nuclear localization and destabilize MYC and ETS1 in an ERK-independent manner. Significantly, targeting ABL1/2 and DDR1 with an FDA-approved anti-leukemic drug, reverses intrinsic MEKi resistance, delays acquisition of acquired resistance, and doubles the survival time in a *NRAS*-mutant mouse model. These data indicate that repurposing FDA-approved drugs targeting ABL1/2 and DDR1 may be a novel and effective strategy for treating patients with treatment-refractory NRAS-driven melanomas.

## 1. Introduction

If caught early, most melanomas are cured (98% five-year survival rate); however, the five-year survival rate dramatically decreases (27%) for patients with distant disease (ACS Facts & Figures 2021). Compared with more common *BRAF*-mutant melanomas (50–70% of cases), the understudied *NRAS*-mutant subtype (15–25% of cases) is more aggressive, and patients have a lower median overall survival [1]. Low patient survival is due to the high melanoma mitotic index, thicker presentation at diagnosis, high incidence of distant metastases, and fewer treatment options [1]. First-line therapy for patients with metastatic *NRAS*-mutant melanomas is immune checkpoint blockade; however, only a subset of patients respond (30–60%) [2,3]. Unlike melanomas harboring *BRAF* mutations, which are highly sensitive to MAPK inhibition (BRAF and MEK inhibitors; BRAFi, MEKi), BRAFi treatment of melanomas harboring wild-type BRAF (including *NRAS*-mutant) activates MEK/ERK and increases melanoma growth [4]. This phenomenon, termed paradoxical ERK activation, is due to activation of CRAF as a result of heterodimerization with drug-bound, wild-type BRAF [4]. *NRAS*-mutant melanomas are also resistant to MEKi and median progression-free survival (PFS) in clinical trials was limited to 2.8–3.25 months at best [5]. Thus, the PFS gain was too modest to warrant FDA approval [5]. Therefore, for patients who cannot tolerate immune-checkpoint blockade or whose melanomas are resistant to their effects, 2nd-line drugs are limited to cytotoxic agents, which are largely ineffective [1,4].

ABL1/2 (a.k.a. c-ABL, ARG) non-receptor tyrosine kinases are most known for their oncogenic roles in leukemia; however, accumulating evidence over the past two decades indicates that they also play critical roles in solid tumors including melanoma [6,7]. During melanoma progression, ABL1/2 drive proliferation, survival, invasion, and metastasis by phosphorylating substrates including the CRK/CRKL adapter proteins, which results in induction of transcription factors and proteases that degrade the extracellular matrix [6,8]. However, the contribution of ABL1/2 to MEKi resistance in *NRAS*-mutant melanomas represents a gap in our knowledge. FDA-approved drugs targeting ABL1/2 have been utilized for decades to treat leukemias driven by activated forms of ABL1 (e.g., BCR-ABL) [8]); therefore, repurposing these agents for treatment-refractory melanomas would be an attractive treatment strategy. 

The DDR1 receptor tyrosine kinase, which is activated by binding fibrillar collagen I and network-forming collagen IV, regulates adhesion of melanocytes to the basement membrane [9,10,11,12]. Conflicting tumor-suppressing and tumor-promoting roles for DDR1 have been documented [13]. Although a number of FDA-approved drugs target both ABL1/2 and DDR1, due to the similarity of their kinase domains, the relationship between ABL1/2 and DDR1 activation has not been previously explored. Moreover, to date, the contribution of DDR1 to NRAS-driven melanoma growth and MEKi resistance has not been investigated. Here, we demonstrate that DDR1 contributes to ABL1/2 potentiation during MEKi resistance, and ABL1/2 and DDR1 activities are necessary and sufficient to drive MEKi resistance. Moreover, cotargeting ABL1/2 and DDR1 decreases BRAF/CRAF stability, increases RAF heterodimerization, reduces coupling of NRAS and ERK to BRAF/CRAF, prevents survival, induces apoptosis, reverses intrinsic resistance and delays the onset of acquired resistance, in vivo. These exciting data indicate that drugs targeting ABL1/2 and DDR1, many of which are FDA-approved, may be effective in conjunction with MEKi for patients with *NRAS*-mutant melanomas, a notoriously hard-to-treat class of patients.

## 2. Materials and Methods

Supplemental Methods are available online with this article.

### 2.1. Reagents

Details for reagents and software/algorithms are in Table S1.

### 2.2. Cell Lines

SK-MEL-2P/MR and SK-MEL-30P/MR cells were cultured in RPMI+glutamine, HEPES, and 10% FBS, while SK-MEL-147P/MR cells were grown in DMEM with the above additives. Cells were cultured at 37 °C and 5% CO_2_. Resistant lines were generated by culturing parental cells in increasing concentrations of trametinib up to 20 nM and were then maintained in trametinib (20 nM).

### 2.3. ABL1/2 Activation

ABL1/2 activities were assessed indirectly by examining phosphorylation of their substrates CRK/CRKL at ABL1/2 phosphorylation sites (pY221, pY207; termed pCRKL). pCRKL is recognized as a reliable read-out of ABL1/2 activities [14,15,16,17,18,19].

### 2.4. Viability Assays

Cells were plated in triplicate or quadruplicate in 96-well plates, drug-treated the following day, and harvested 72–96 h later using CellTiter Glo (see manufacturer’s instructions, Table S1). Trametinib was removed from resistant cells two days prior to plating in order to be able to assess the effects of ABL/DDR inhibitors in the absence or presence of trametinib. To measure trypan blue exclusion, cells were plated in 60 mm dishes, drug-treated for 96 h, trypsinized, and the percentage of live cells were scored by diluting cells 1:1 in trypan blue (Biorad, Hercules, CA, USA) and counting on a TC-20 automated cell counter (Biorad, Hercules, CA, USA).

### 2.5. Apoptosis Assays

Cells were drug-treated (24–96 h) the day after plating. Detached/attached cells were lysed (RIPA buffer: 50 mM Tris pH 7.5, 150 mM NaCl, 1% Triton-X 100, 0.1% SDS, 1% sodium deoxycholate, 1 mM PMSF, 1 mM sodium orthovanadate, 25 mM sodium fluoride, and 10 μg/mL leupeptin, aprotinin, and pepstatin), and the lysate was used for Western blots.

### 2.6. Clonogenic Assays

Cells were plated in 6-well dishes, drug-treated (see Figure legends for treatment times), washed, incubated for an additional 4–6 days, fixed (50% methanol), and stained (0.05–0.1% crystal violet in 50% methanol). The plates were imaged on a GE Healthcare AI600, and colonies/staining were quantitated with the ImageJ/ColonyArea Plug-In [20].

### 2.7. SiRNA Transfection

Cells were transfected with scrambled or specific siRNAs (5–10 nM) using Lipofectamine 2000 (see Table S1; see manufacturer’s instructions), and lysed 72 h later in RIPA buffer.

### 2.8. Coimmunoprecipitation (coIP)

Cells were lysed (TNEN buffer: 50 mM Tris, pH7.5, 50 mM NaCl, 2 mM EDTA, 0.5% NP-40, + inhibitors—see above), and the lysate was precleared with mouse IgG and protein G sepharose (1 h). Precleared lysates were incubated with agarose-conjugated antibodies (2.5–5 μg/500–800 μg in 1.2 mL) or normal mouse IgG (5 μg) for 3 h. IPs were washed three times in lysis buffer, the complexes were run on SDS-PAGE gels, and the membranes were blotted.

### 2.9. Xenograft Assays

SK-MEL-147 parental (3 × 10^6^) cells, diluted 1:1 with high concentration matrigel (Table S1), were injected subcutaneously into 6-week-old female, nude mice (NU/J; JAX; Bar Harbor, Maine). When tumors were 150–200 mm^3^, the mice were randomized and treated with vehicle (0.5% hydroxypropyl methylcellulose (HPMC) + 0.05% Tween 80) [21], nilotinib (33 mg/kg, b.i.d., o.g.), trametinib (1 mg/kg, q.d., o.g.), or trametinib+nilotinib. Since no toxicity was noted, nilotinib was increased to 50 mg/kg on d12. Tumors were measured three times weekly with linear calipers, and the volumes were calculated (LxW^2^/2). Mice were euthanized when tumors reached 1500 mm^3^ or became ulcerated. The experiments were performed under IACUC protocol #2020–3426 (approval 6 November 2021) in accordance with University and NIH guidelines. These guidelines include: (a) using alternatives to animals, when possible (replacement); (b) using only the number of animals needed to achieve statistical significance (reduction); and (c) refining the experimental conditions to minimize pain and discomfort (refinement).

### 2.10. Statistics

In vitro studies were analyzed with one-way ANOVA (>two comparisons), two sample (comparisons between treatment groups), or one-sample *t*-tests (comparisons against normalized controls) using the Holm’s method for multiple comparisons adjustment for one- and two-sample tests and Bonferroni multiple comparisons test for one-way ANOVA. In vivo studies were analyzed with the linear mixed model (SAS, v9.4) to compare log-transformed tumor volumes for trametinib and trametinib+nilotinib groups. Kaplan–Meier and logrank tests (R, v4.0.0) were used for comparing time to tumor doubling. In all cases, two-tailed *p* values were reported (*p* < 0.05 was considered statistically significant).

## 3. Results

### 3.1. ABL1/2 Are Activated during MEKi Resistance

**Figure 1:**
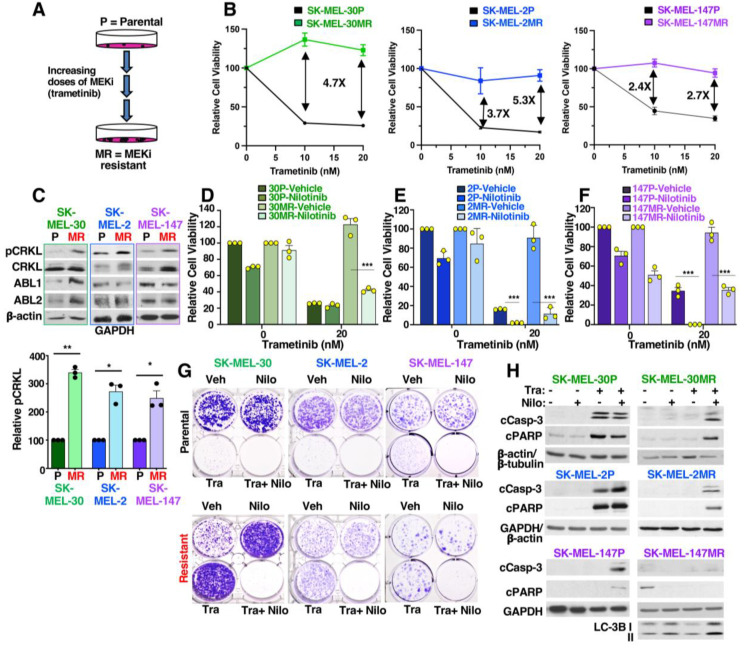
ABL1/2 are activated during acquired MEKi resistance, and drugs targeting ABL1/2 reverse intrinsic and acquired resistance, and induce apoptosis. (**A**) Establishment of resistant cell lines. Parental (P) cells harboring mutant NRAS were incubated with increasing concentrations of trametinib until the lines were resistant to 20 nM (-MR). (**B**) Cell viability assays (CellTiter Glo-CTG). Parental (P) and resistant (MR) cells were treated for 72 h (SK-MEL-30, SK-MEL-2) or 96 h (SK-MEL-147) with the indicated trametinib doses. Mean ± SEM for n = 3 independent experiments. Arrows/numbers indicate the fold difference in trametinib sensitivity between parental and resistant cell lines. (**C**) ABL1/2 kinase activities were indirectly assessed by Western blot analysis using an antibody that recognizes the ABL1/2 phosphorylation sites on substrates CRK/CRKL (termed pCRKL), a well-accepted read-out of ABL1/2 activities [14,15,16,17,18,19]. Quantitation for n = 3, mean ± SEM is shown. * *p* < 0.05 (left→right; *p* = 0.016, 0.028), ** *p* = 0.002. (**D**–**F**) Cell viability assays (CTG) using cells treated with vehicle, trametinib, nilotinib (5–6 μM), or the combination (72 h). Three doses of each drug were utilized; additional doses are shown in Figure S1. Mean ± SEM for n = 3. *** *p* < 0.001. Actual *p*-values (left→right): (**D**): 0.00032; (**E**): *p* < 0.0001, 0.000726; (**F**): 0.000101; 0.000627. (**G**) Clonogenic (colony) assays. Cells were treated with trametinib (Tra; 20 nM) and/or nilotinib (5 μM for SK-MEL-147; 2.5 μM for other two lines) for 7 days, washed, incubated without drugs for an additional 4 (SK-MEL-2, SK-MEL-147) or 6 (SK-MEL-30) days, and then the colonies were stained with crystal violet. Images are representative of n = 3–4. (**H**) Western blot analysis using attached and detached cells following treatment with nilotinib (Nilo, 2.5 μM) and/or trametinib (Tra, 20 nM) for 24 h (SK-MEL-2P, SK-MEL-2MR), 48 h (SK-MEL-30P, SK-MEL-30MR, SK-MEL-147P), or 96 h (SK-MEL-147MR). Results are representative of n = 3. cCasp-3 = cleaved caspase-3; cPARP = cleaved PARP. The uncropped blots are shown in File S1.

To understand why melanoma cells harboring mutant forms of *NRAS* are resistant to MEKi, which are effective for treating *BRAF*-mutant melanomas, we established polyclonal cells with acquired trametinib resistance from cell lines harboring *NRAS* mutations (SK-MEL-2, Q61R; SK-MEL-30, Q61K; SK-MEL-147, Q61R; Figure 1A). Parental lines are denoted with a “P” (e.g., SK-MEL-2P), whereas long-term, acquired resistance cell lines are denoted with “MR” (e.g., SK-MEL-2MR). Parental SK-MEL-30P and SK-MEL-2P cells are highly sensitive to trametinib (MEKi), whereas SK-MEL-147P parental cells harbor some intrinsic resistance (Figure 1B; 50% survival in 10–20 nM trametinib). In contrast, acquired resistance (MR) cell lines are completely insensitive to the effects of trametinib (Figure 1B). Since ABL1/2 play important roles in driving melanoma progression and are activated by ERK pathway components (BRAF, ERK) in BRAF-mutant melanomas [22], we examined whether their activities are elevated during MEKi resistance in NRAS-driven melanomas. Since commercially available phospho-specific ABL1/2 antibodies are not sensitive enough to assess endogenous ABL1/2 activities in solid tumor cell lines, we indirectly measured ABL1/2 activities by Western blot using an antibody that recognizes the ABL1/2 phosphorylation sites on substrates CRK/CRKL (pY221/pY207; termed pCRKL). Phosphorylation of CRK/CRKL has been shown to be a reliable read-out of ABL1/2 activity [14,15,16,17,18,19]. Importantly, we found that phosphorylated CRKL was elevated in all resistant lines (Figure 1C).

### 3.2. Targeting ABL1/2 Reverses Intrinsic and Acquired Trametinib Resistance

To examine whether ABL1/2 activation is required for intrinsic and acquired MEKi resistance, we treated cells with the second generation, ATP-competitive ABL1/2 inhibitor, nilotinib. On its own, nilotinib was modestly effective at reducing the viability of parental cells, but was less efficient in matched resistant SK-MEL-2MR and SK-MEL-30MR cell lines (Figure 1D–F and Figure S1A–C). In contrast, nilotinib alone was effective in reducing the viability of SK-MEL-147MR resistant cells (Figure 1F and Figure S1C). In combination with trametinib, nilotinib dramatically reduced survival, and reversed intrinsic (SK-MEL-147P), and acquired trametinib resistance (MR lines) as assessed using short-term viability (Figure 1D–F and Figure S1A–C) and long-term clonogenic (Figure 1G) assays. Moreover, the loss of viability was permanent as illustrated by induction of apoptosis via caspase-3 and PARP cleavage blots in parental lines and in SK-MEL-2MR and SK-MEL-30MR (Figure 1H). Interestingly, while nilotinib and nilotinib+trametinib dramatically reduced SK-MEL-147MR viability and percentage of live cells (Figure 1F and Figure S1D), we did not observe cleaved PARP or caspase-3 in combination-treated cells (Figure 1H). These data indicate that the mechanism by which nilotinib induces cell death in this cell line does not involve apoptosis but rather is via another form of cell death. Indeed, combination treatment increased LC-3B II, an indicator for the presence of autophagosomes (Figure 1H) [23].

Peeper and colleagues showed that some melanoma cells treated with BRAF inhibitors become “addicted” to the drugs, such that drug withdrawal induces cell death [24]. Interestingly, SK-MEL-30MR, but not the other resistant cell lines, appeared to be addicted to trametinib, as cells grew much better in trametinib as compared to vehicle (Figure 1G). Interestingly, a similar effect was also observed with nilotinib indicating that nilotinib and trametinib likely impact the same pathway (e.g., ERK2) to drive drug addiction [24].

### 3.3. ABL1/2 and DDR1 Drive Acquired MEKi Resistance

**Figure 2:**
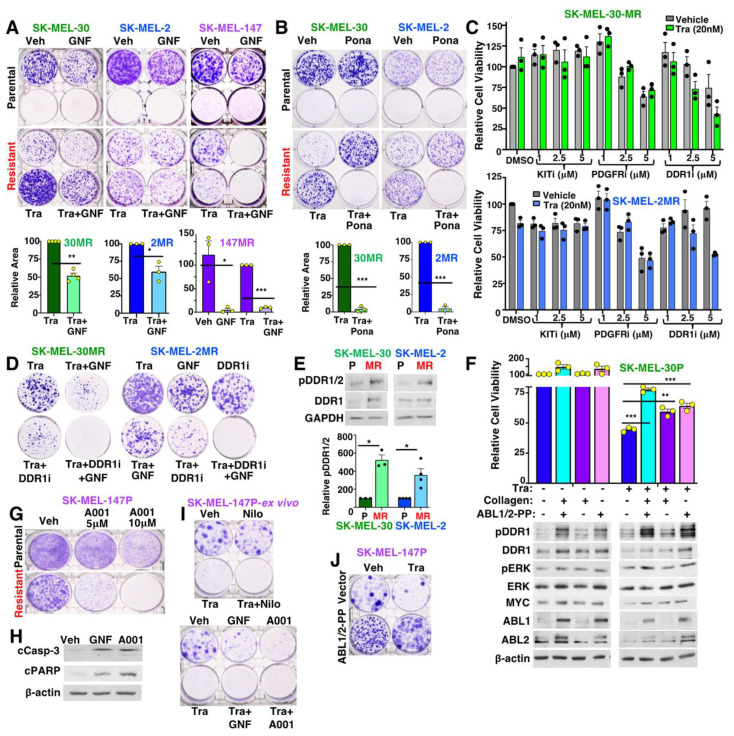
ABL1/2 and DDR1 are required for MEKi resistance in SK-MEL-2MR and SK-MEL-30MR. (**A**,**B**,**D**,**G**) Clonogenic assays. Plated cells were drug-treated for 7 days (**A**: SK-MEL-147MR; **B**,**D**,**G**) or 14 days (**A**: other cell lines) and 7 day treatment wells incubated without drugs for an additional 4 days. Drug doses are as follows: trametinib (Tra; 20nM), GNF-5 (GNF; 12.5 μM), ponatinib (pona, 100 nM), DDR-IN-1 (DDR1i, 2.5 μM), and ABL001 (A001, indicated doses. Quantitation indicates mean ± SEM for n = 3 independent experiments. * *p* < 0.05, ** *p* < 0.01, *** *p* < 0.001. Exact *p*-values (left→right): 0.0011, 0.037, 0.016, 0.000125, 0.0007, 0.00069 using single sample *t*-tests. (**C**) CellTiter Glo (CTG) viability assays (72 h) using resistant lines treated with specific inhibitors targeting KIT/CSF (PLX3397, KITi), DDR1 (DDR-IN-1, DDR1i), or PDGFR (CP673451, PDGFRi) −/+ trametinib (20 nM). Mean ± SEM for n = 3. (**E**,**H**) Western blots using parental or resistant cell lysates (**E**) or lysates from resistant cells treated with GNF-5 or ABL001 (A001, 10 μM) for 96 h (using attached and detached cells, (**H**)). (**E**) Mean ± SEM, n = 3 * *p* < 0.05 (0.016, 0.03; left→right). cCasp-3 = cleaved caspase-3; cPARP=cleaved PARP. (**F**) Cells expressing vector or activated forms of ABL1 and ABL2 (PP) were plated on tissue culture plates (−) or collagen I-coated plates (+) and treated with vehicle (−) or trametinib (+, 0.75 nM) for 72 h (top) or 18 h (bottom), followed by viability assay (CTG, top) or Western blot (bottom). Mean ± SEM, n = 3. One-way ANOVA *p* < 0.0001. Bonferroni’s multiple comparison tests were used to compare each treatment group to vector/no collagen-trametinib-treated cells. *p*-values (left→right): <0.0001, 0.001, 0.0001. (**I**,**J**) Clonogenic assays using cells from a SK-MEL-147P xenograft that developed trametinib resistance, in vivo (**I**) or parental cells stably expressing vectors or ABL1/2-PP, treated with trametinib (2.5 nM) for 3d, and incubated without drugs for 18d (**J**). Drug doses are as folows: trametinib (Tra, 20 nM), nilotinib (2.5 μM), GNF-5 (GNF, 12.5 μM), ABL001 (A001, 10 μM). Results are representative of n = 3. The uncropped blots are shown in File S1.

To identify the nilotinib target(s) that drive acquired resistance, we first examined whether treatment with a highly specific, allosteric ABL1/2 inhibitor (GNF-5; exclusively targets ABL1/2) [8], phenocopies nilotinib’s effects. If nilotinib reverses resistance solely by targeting ABL1/2, then GNF-5 should mimic the effects of nilotinib. SK-MEL-2MR and SK-MEL-30MR cells were relatively insensitive to GNF-5 on its own, similar to their response to nilotinib (Figure 2A). In the presence of trametinib, GNF-5 inhibited colony formation but only by ≅50%, indicating that it is much less efficient than nilotinib (95–98% reduction; Figure 2A). Similar effects were observed with a second highly specific, allosteric BCR-ABL inhibitor, ABL001/asciminib [21] (Figure S1E). These data contrast with those obtained with SK-MEL-147MR cells as GNF-5 was extremely efficient (90–95%) at inhibiting colony formation in the presence of trametinib, mimicking nilotinib in this cell line (Figure 2A). Moreover, GNF-5 was also highly effective in the absence of trametinib in SK-MEL-147MR cells (Figure 2A). Since the allosteric inhibitors are less sensitive than nilotinib, they require higher doses to inhibit ABL1/2; however, the doses utilized here are in-range with those used by other groups [17,18]. Taken together, these data indicate that nilotinib’s effects in SK-MEL-2MR and SK-MEL-30MR are likely only partially mediated by ABL1/2 whereas in SK-MEL-147MR, nilotinib likely solely acts by targeting ABL1/2.

In order to identify the second nilotinib target involved in trametinib resistance in SK-MEL-2MR and SK-MEL-30MR, we treated the lines with another ATP-competitive ABL1/2 inhibitor (ponatinib) that has a different set of non-ABL targets. Interestingly, ponatinib’s effects were identical to nilotinib (Figure 2B), indicating that targets in common between nilotinib and ponatinib drive resistance. In addition to ABL1/2, nilotinib and ponatinib also inhibit KIT, CSF1R, PDGFR, and DDR1 [8]. To identify which of these four kinases is involved in MEKi resistance in SK-MEL-2MR and SK-MEL-30MR, we utilized specific inhibitors for each molecule. KIT/CSF1R inhibition (PLX3397) had no effect on cell viability, whereas PDGFR (CP673451) blockade reduced viability but did not cooperate with trametinib (Figure 2C). In contrast, DDR1 inhibition (DDR-IN-1) reversed trametinib resistance and cooperated with GNF-5 (ABL1/2 inhibitor; Figure 2C,D), similar to nilotinib which targets both DDR1 and ABL1/2. Consistent with a role for DDR1 in driving resistance in SK-MEL-2MR and SK-MEL-30MR, DDR1 was activated in resistant cells (Figure 2E). DDR1 is activated by binding collagen I and IV and stimulates collagen IV synthesis [9,10,12]. Indeed, RNA sequencing (RNA-seq) demonstrated that collagen I and collagen IV subunits were upregulated in acquired resistance cells (SK-MEL-2MR: *COL1A1*, *COL1A2*, *COL4A2*, *COL4A5*, *COL4A6*; SK-MEL-30MR: *COL1A1*, *COL4A5*; Dataset S1). Moreover, GSEA analysis identified upregulation of numerous collagen synthesis pathways in resistant lines (Figure S2). Thus, DDR1 is activated in SK-MEL-2MR and SK-MEL-30MR, in the absence of external collagen stimulation, likely due to collagen upregulation and secretion by the melanoma cells themselves. Moreover, activation of ABL1/2 and DDR1 is required for MEKi resistance. Consistent with these conclusions, DDR1 was activated following plating of parental cells on collagen I (Figure 2F-bottom), which increased cell viability in the presence of trametinib, and cooperated with activated forms of ABL1/2 (PP) [25] to induce trametinib resistance (Figure 2F and Figure S1F).

### 3.4. SK-MEL-147MR Are Exquisitely Dependent on ABL1/2

In contrast to SK-MEL-2MR and SK-MEL-30MR, which are insensitive to nilotinib or GNF-5 in the absence of trametinib, the survival of SK-MEL-147MR cells was permanently abrogated by GNF-5 and ABL001 in the absence or presence of trametinib (Figure 2A-right,G,H). Importantly, drugs targeting ABL1/2 (nilotinib, GNF-5, ABL001) also reduced the viability of SK-MEL-147P cells that develop trametinib resistance in vivo, demonstrating that the data are clinically relevant (Figure 2I). To confirm these findings, we attempted to silence ABL1/2 with an shRNA that targets both proteins. Unfortunately, even when using an inducible system (IPTG), we were unable to stably silence ABL1/2 in SK-MEL-147MR as the clones either died or re-expressed ABL1/2. These data are consistent with the notion that SK-MEL-147MR cells are highly dependent on ABL1/2 as even low-level loss of ABL1/2 (leaky inducible system) was not compatible with survival. Importantly, expression of constitutively active forms of ABL1/2 (PP) into parental SK-MEL-147P cells was sufficient to increase survival and promote trametinib resistance, driving cells that had partial intrinsic resistance into an acquired resistance phenotype (Figure 2J). Thus, acquired MEKi resistance in SK-MEL-147MR cells appears to be driven solely by ABL1/2 unlike SK-MEL-2MR and SK-MEL-30MR, which require cooperation of ABL1/2 and DDR1.

### 3.5. DDR1 and CRAF→ERK Contributes to ABL1/2 Activation during Resistance

**Figure 3:**
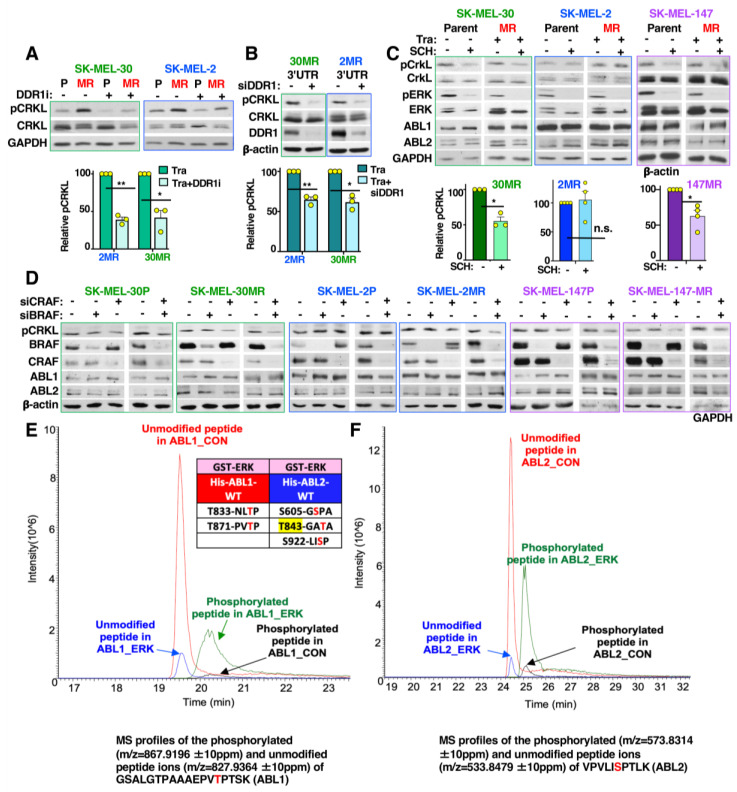
CRAF/ERK and DDR1 contribute to ABL1/2 activity potentiation during acquired MEKi resistance. (**A**,**B**) Phospho-CRKL expression, a reliable read-out of ABL1/2 activities [14,15,16,17,18,19], was assessed by Western blotting of lysates from parental (P) and/or resistant (MR) cells treated with vehicle or DDR1 inhibitor, DDR-IN-1 (4 μM; DDR1i), for 24 h (**A**) or transfected with scrambled or DDR1 siRNA targeting the 3’UTR (**B**). Results with a second siRNA are shown in Figure S3A. Quantitation is mean ± SEM, n = 3. (**A**): * *p* = 0.026, ** *p* = 0.0032. (**B**): * *p* = 0.017, ** *p* = 0.0092. (**C**,**D**) Phospho-CRKL expression was assessed in lysates from cells treated with trametinib (20 nM) in the absence or presence of the ERK inhibitor SCH772984 (0.1 μM) for 24 h (**C**) or transfected with scrambled (−), BRAF, or CRAF (5 nM) siRNA for 72 h (**D**). (**C**) Quantitation is mean ± SEM, n = 3−4. 30 MR, * *p* = 0.016. 147 MR, * *p* = 0.019. Modulation of ABL1/2 activities (pCRKL) following ARAF knockdown is shown in Figure S3B. (**E**,**F**) Mass spectrometry results for recombinant ERK2 phosphorylation of recombinant ABL1 and ABL2, in vitro. Table Inset: ERK2 phosphorylation sites in ABL1b and ABL2. Yellow highlight indicates a putative 14-3-3 binding site (14-3-3 Pred program) [17,30]. MS profiles of phosphorylated and unmodified peptide ions for GSALGTPAAEPVTPTSK on ABL1 (left) and VPVLISPTLK on ABL2 (right). Additional mass spectrometry profiles are provided in Figure S3C,D. The uncropped blots are shown in File S1.

Since ABL1/2 activities were elevated and contributed to trametinib resistance in all lines, we investigated the mode of potentiation. Previously, we found that ABL kinases are activated by receptor tyrosine kinases such as EGFR, PDGFR, and IGF1R [26,27,28], and BRAF contributes to ABL1/2 activation in melanoma cells harboring BRAF-V600E [22]. Thus, we tested whether RAF proteins or DDR1 (a receptor tyrosine kinase) are involved in ABL1/2 activation in *NRAS*-mutant cells during MEKi resistance. Interestingly, silencing or inhibiting DDR1 (in SK-MEL-2MR and SK-MEL-30MR), ERK inhibition, or silencing CRAF (but not BRAF or ARAF) reduced CRKL (ABL1/2 substrate) phosphorylation on ABL1/2 sites, a reliable readout of ABL1/2 activity (Figure 3A–D and Figure S3A,B) [14,15,16,17,18,19]. Since DDR1 and CRAF activate ERK [13], and we previously showed that ERK phosphorylates ABL1/2 [22], we mapped the ERK phosphorylation sites on ABL1/2. Interestingly, ERK phosphorylated a consensus ERK phosphorylation site in ABL1 (ABL1b-T871; 1a and 1b are isoforms with alternatively spliced 1st exons [8]). ERK also phosphorylated a site that resides nine amino acids upstream of a putative 14-3-3 binding site (ABL1b-T833). In ABL2, ERK phosphorylated a predicted 14-3-3 binding site (ABL2-T843, yellow highlight; Figure 3E,F and Figure S3C,D). While 14-3-3 binding to ABL1 is known to sequester it in the cytoplasm, which facilitates its cytoplasmic activation and transforming ability [29], this is the first demonstration of phosphorylation of a predictive 14-3-3 binding site in ABL2.

### 3.6. MEKi-Resistant Cells Utilize Diverse Mechanisms to Activate ERK/MYC/ETS1/RSK1 Signaling

**Figure 4:**
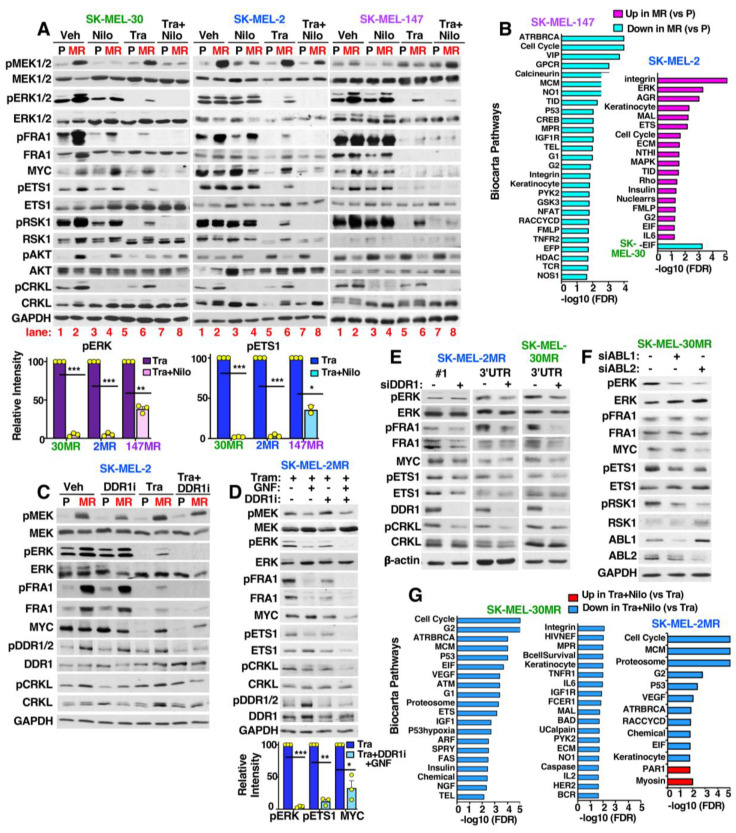
ABL1/2 and DDR1 are required for ERK/MYC/ETS1/RSK1 reactivation during acquired resistance. (**A**,**C**,**D**) Western blots from cells treated with drugs or vehicle (Veh; DMSO) for 24 h (**A**) or 48 h (**D**). Drug doses are as follows: trametinib (Tra; **A**,**C:** 20 nM; **D:** 10 nM), nilotinib (Nilo; 2.5 μM), DDR-IN-1 (DDR1i, 4 μM-**C** or 2.5 μM-**D**).Experiments with GNF-5 (**D**) used lower doses of DDR1i and trametinib and a longer time (48 h) in order to increase the efficiency of GNF-5-mediated inhibition of ABL1/2. A second cell line is shown in Figure S4A,B. Graphs are mean ± SEM. (**A**) pERK: *** *p* < 0.0001, ** *p* = 0.008; pETS1: *** *p* < 0.0001, * *p* = 0.039. (**D**) *** *p* < 0.0001, ** *p* = 0.003, * *p* = 0.027. (**B**,**G**) RNA-seq/GSEA comparing resistant to parental cells (**B**) or trametinib-treated resistant lines to trametinib+nilotinib (**G**). (**B**) Only one Biocarta pathway was changed for SK-MEL-30MR. (**G**) Trametinib + nilotinib did not induce Biocarta pathway changes in SK-MEL-147MR. Data is shown in Figures S2 and S5 and Datasets S1,S3. (**E**,**F**) Western blots from lines transfected with DDR1 (10 nM) or ABL1/2 (20 nM) siRNAs for 72 h. Blots are representative of n = 3. A second line is shown in Figure S4C. pCRKL blots for Figure 4C,E are from the same experiment as Figure 3A,B and Figure S3A. The uncropped blots are shown in File S1.

To identify the mechanism of MEKi resistance, we first assessed activation of the ERK and AKT pathways, since NRAS activates both pathways in *NRAS*-mutant melanoma cells [3]. In parental cells, ERK activation and subsequent activation of ERK nuclear (FRA1, MYC) or cytoplasmic (ETS1, RSK1) downstream targets was efficiently inhibited by trametinib. In contrast, in all three acquired resistance cell lines, ERK/MYC/ETS1/RSK1 remained induced/activated in the presence of trametinib (Figure 4A; compare lanes 1,5 with 2,6). AKT phosphorylation was upregulated in SK-MEL-30MR, whereas in the other two resistant lines, pAKT was decreased (Figure 4A; lanes 1,2 and 5,6). Thus, ERK/MYC/ETS1/RSK1 activation is important for resistance in all lines, and PI3K/AKT may contribute to SK-MEL-30MR resistance.

To gain further insight into how acquired resistance lines activate ERK/MYC/ETS1/RSK1 signaling, we performed whole exome sequencing, identifying acquired mutations in trametinib-resistant cell lines. SK-MEL-147MR cells harbored a mutation in MEK1 (MAP2K1-F129L; Table S2 and Dataset S2) that resides within the allosteric, trametinib-binding pocket [31]. In SK-MEL-30MR and SK-MEL-2MR, no ERK pathway mutations were identified. However, SK-MEL-2MR cells harbored frameshift and splice site mutations (likely loss-of-function) within the ephrin (ligand)-binding domain of EPHA4 (N140fs, W54L; Table S2 and Dataset S2). EPHA4 downregulation increases pERK, proliferation, migration, and metastasis in melanoma, and is inversely correlated with overall survival [32]. Finally, SK-MEL-30MR cells acquired a mutation in GNAI1 (G125E; Table S2 and Dataset S2), a Giα1 subunit of heterotrimeric GTPases which is known to activate ERK [33].

We also utilized RNA-seq to identify mRNA changes in acquired resistance cells. In SK-MEL-2MR, GSEA/Biocarta pathway analysis showed upregulation of pathways involved in cell cycle progression (ERK, EGR, ETS, G2, MAPK, insulin, EIF, IL6, and keratinocyte) and invasion/metastasis (MAL, integrin, ECM, RHO, and AGR; Figure 4B and Figure S2, Dataset S1). The affected genes included RTKs (*EGFR*, *MUSK*, *NGFR*, *FGFR1*, *PDGFRB*), growth factors (*VEGFRA/C*; *PDGFA/B/C/D*; *FGF1, 2, 12, 16*; *HB-EGF*; *NGF*), and RAS/RAF (*RRAS1/2*, *NRAS*, *ARAF*, *BRAF*, *CRAF*; Dataset S1). In contrast, SK-MEL-30MR had few upregulated growth factors or growth factor receptors (only *FGFR4* and *FGF2, 7, 12, 14*). Moreover, *BRAF* was very significantly upregulated (Log2FC = 3.4; FDR = 1.2 × 10^−46^; Dataset S1), but other *RAS*/*RAF* mRNAs were not significantly changed. Furthermore, only one Biocarta geneset was altered in SK-MEL-30MR (Figure 4B and Figure S2), although using the KEGG database, we identified upregulation of pathways involved in metabolism, extracellular matrix degradation, and downregulation of proliferative pathways (Figure S2, and Dataset S1). Unlike SK-MEL-2MR and SK-MEL-30MR, numerous Biocarta pathways were downregulated in SK-MEL-147MR cells including those controlling proliferation, DNA repair, and DNA replication (Figure 4B and Figure S2, Dataset S1). In summary, acquired resistance in SK-MEL-2MR is associated with global upregulation of RTK and RAS/RAF mRNAs and the cell line harbors an EPHA4 mutation. SK-MEL-30MR resistance is associated with highly upregulated BRAF mRNA and G-protein-coupled receptor mutations. Finally, SK-MEL-147MR contains a MEK1 mutation and downregulation of proliferation/survival pathways likely mediated by changes in G1/G2 checkpoints, DNA repair, and DNA replication.

### 3.7. ABL1/2 and DDR1 Are Critical Nodes Mediating ERK/MYC/ETS1/RSK1 Activation during Resistance

To identify the mechanism by which ABL1/2 and DDR1 drive acquired resistance, we first examined the effect of nilotinib on signaling. Targeting ABL1/2 and DDR1 with nilotinib abolished or dramatically reduced trametinib-induced ERK/MYC/ETS1/RSK1 activation in SK-MEL-2MR and SK-MEL-30MR, cell lines in which resistance is driven by ABL1/2 and DDR1 (Figure 4A, lanes 8 vs. 6). Nilotinib also cooperated with trametinib to inhibit ERK/MYC/ETS1/RSK1 activation in SK-MEL-147MR, which relies mainly on ABL1/2 for trametinib resistance (Figure 4A, lanes 8 vs. 6). Furthermore, DDR1-specific inhibition (DDR-IN-1, DDR1i) in SK-MEL-2MR and SK-MEL-30MR reduced pERK/MYC, and cooperated with the ABL1/2-specific inhibitor, GNF-5 (Figure 4C,D and Figure S4A,B). Thus, DDR-IN-1+GNF-5 mimics the effects of nilotinib. Similarly, silencing DDR1 or ABL1/2 also reduced ERK/MYC/ETS1/RSK1 signaling (Figure 4E,F and Figure S4C). Thus, cotargeting ABL1/2 and DDR1 reversed resistance regardless of the mechanism of ERK/MYC/ETS1/RSK1 reactivation, and had little/no effect on pAKT (Figure 4A).

To gain additional insight into how nilotinib reverses acquired trametinib resistance, we performed RNA-seq comparing resistant cells treated with trametinib to the same cells treated with trametinib+nilotinib. Since nilotinib reverses resistance in SK-MEL-2MR and SK-MEL-30MR by targeting ABL1/2 and DDR1, we identified changes in common in the two lines. The expression of many ERK targets and regulators (*FOS*, *FRA1-FOSL1*, *ETV4/5*, *EGR1*, *DUSPs*, *SPRYs*, *MAFF*, *EPHA2*, *HMGA2*, *KCCN4*, *UBALD2*, *NEDD9*) [34,35,36,37,38,39,40] were reduced by nilotinib as were cell cycle genes (*CCND1*-cyclin D1, *CDC25*, *CDKN2D*, *PCNA*), whereas the *PRUNE2* tumor suppressor was upregulated (Table S3 and Dataset S3) [41]. Moreover, nilotinib induced downregulation of many Biocarta pathways including Cell Cycle, G2 checkpoint, Proteosome, and EIF (Figure 4G and Figure S5, Dataset S3). A small subset of the ERK targets and regulators impacted by nilotinib in SK-MEL-2MR and SK-MEL-30MR were also changed in SK-MEL-147MR, but there were also unique ERK-regulated targets (Figure S5B, Table S3, and Dataset S3). Moreover, no Biocarta pathways were significantly changed in SK-MEL-147MR treated with trametinib+nilotinib, although using the KEGG database, we found the following genesets/pathways to be upregulated: apoptosis, autophagy (consistent with our data in Figure 1H), adherens and tight junctions, actin cytoskeleton, and ubiquitin proteolysis (Figure S5B, Table S3, and Dataset S3). Furthermore metabolism, DNA replication, and DNA repair were downregulated (Figure S5B, Table S3, and Dataset S3). Thus, cotargeting ABL1/2 and DDR1 not only prevents ERK activation of downstream targets but also impacts other pathways involved in proliferation, survival, and melanoma progression.

### 3.8. Targeting ABL1/2 and DDR1 Alters Expression of RAF, MYC and p27/KIP1 and Induced RAF Heterodimerization

**Figure 5:**
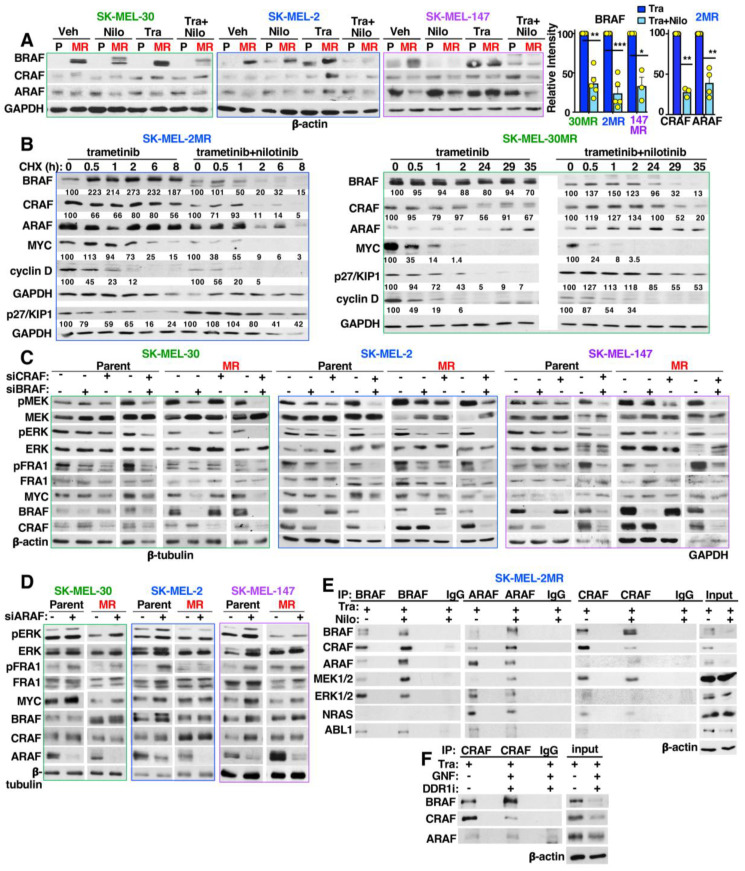
Targeting ABL1/2 and DDR1 promotes RAF heterodimerization and degradation in trametinib-resistant cells. (**A**) Western blots using cells treated with vehicle (Veh), trametinib (Tra, 20 nM), and/or nilotinib (Nilo, 2.5 μM, 24 h). Mean ± SEM, n = 3–5; * *p* < 0.05, ** *p* ≤ 0.01, *** *p* < 0.001. Exact *p*-values (left→right): 0.0011, 0.0008, 0.03, 0.004, 0.006. (**B**) Protein stability assays. Cells were drug-treated for 24 h followed by cycloheximide treatment (CHX, 100 μM). Quantitation relative to GAPDH is shown below the blots, and quantitation for n = 3 can be found in Figure S6B–K. For SK-MEL-30MR, blots are contiguous: white bar removes an unnecessary lane. (**C**,**D**) Western blots using cells transfected for 72 h with scrambled (-), BRAF, CRAF (**C**), or ARAF (**D**) siRNA (5 nM). Blots are representative of n = 3. Some controls are the same as in Figure 3D since they are from the same replicate. (**E**,**F**) Coimmunoprecipitations (coIPs). RAF isoforms were IP’d from treated cells from (**A**) ((**E**), 24 h), or from cells treated with GNF-5 (12.5 μM) + DDR1i (2.5 μM) (**F**), and subjected to Western blot. To increase the efficiency of ABL1/2 inhibition by GNF-5, cells were treated for 48 h using a lower DDR1i dose. IgG is the isotype negative control IP. CoIPs for a second cell line are shown in Figure S6L. Representatives of n = 2–3 independent experiments are shown. The uncropped blots are shown in File S1.

MEK phosphorylation by upstream kinases can reduce the affinity of trametinib to the allosteric site and drive resistance [42]. MEK may be activated downstream of EPHA4 or GNAI1 in SK-MEL-2MR and SK-MEL-30MR, respectively, and/or by increased BRAF (both lines), CRAF (SK-MEL-2MR), ARAF (SK-MEL-2MR) and/or NRAS (SK-MEL-2MR) expression (Table S2, Dataset S1 and S2). Indeed, BRAF protein was increased in all resistant lines, and CRAF and ARAF were also induced in SK-MEL-2MR, particularly in the presence of trametinib (Figure 5A). Interestingly, parental and resistant SK-MEL-30 cells harbor class III, non-V600 BRAF mutations (E257K, D287H; Dataset S2, last tab), which require cooperation with activated RAS for full transforming activity [43]. Importantly, nilotinib (+trametinib) induced a BRAF mobility shift and decreased BRAF expression in all resistant lines (Figure 5A). Nilotinib also reduced CRAF (SK-MEL-2MR) and ARAF protein (SK-MEL-2MR, SK-MEL-147MR; Figure 5A). RNA-seq indicated that nilotinib (+trametinib) did not reduce *RAF* mRNAs (Dataset S3; FDR >>> 0.05; CRAF gene is RAF1), which was confirmed by qPCR (Figure S6A). Rather, nilotinib dramatically reduced the half-life of BRAF protein as BRAF has a half-life of 11.75 h ± 2.56 in trametinib-treated SK-MEL-2MR cells whereas the half-life was reduced to 2.5 h ± 0.76 in the same cells treated with trametinib+nilotinib (Figure 5B and Figure S6B, n = 3). Nilotinib-mediated reduction in BRAF stability was also observed in SK-MEL-30MR, which harbored a highly stable BRAF protein (Figure 5B and Figure S6C). Moreover, nilotinib also accelerated degradation of CRAF (both cell lines) and ARAF (SK-MEL-2MR) (Figure 5B and Figure S6D–G), but did not impact ARAF stability in SK-MEL-30MR and actually increased ARAF mRNA (Figure S6A,B). Interestingly, nilotinib reduced *MYC* mRNA and MYC protein stability in SK-MEL-2MR, but not in SK-MEL-30MR cells (Figure 5B and Figure S6A,H,I). Nilotinib did not inhibit cyclin D stability in either cell line (and even increased its stability in SK-MEL-30MR), likely because it dramatically reduced cyclin D expression at the mRNA level (*CCND1*; Dataset S3, Figure 5B). Nilotinib also increased the mRNA expression of the cell cycle inhibitor, p27/KIP1 (*CDKN1B*), as assessed by qPCR; Figure S6A), and stabilized p27/KIP1 protein in both cell lines (Figure 5B and Figure S6A,J,K). Thus, nilotinib-mediated inhibition of ERK/MYC/ETS1/RSK1 and resistance is associated with reduced BRAF/CRAF stability, decreased MYC mRNA expression and stability (in some cells), and induction of p27/KIP1 mRNA and stabilization of p27/KIP1 protein.

BRAF and CRAF are both required to drive ERK activation in mutant *NRAS*-dependent melanomas [44]; however, the role of the various RAF proteins during resistance is unknown. ERK pathway activation requires both BRAF and CRAF in all three parental *NRAS*-mutant cell lines as well as in acquired resistant SK-MEL-2MR cells. However, SK-MEL-30MR cells shifted towards relying solely on BRAF, while SK-MEL-147MR shifted towards relying solely on CRAF (Figure 5C). Interestingly, silencing ARAF either did not impact (SK-MEL-2MR, SK-MEL-147MR) or even increased ERK signaling (parental and SK-MEL-30MR; Figure 5D).

Wild-type RAF isoforms function as homo- and heterodimers, and BRAF/CRAF heterodimers drive ERK signaling downstream of mutant *NRAS* in MEKi-sensitive melanoma cells [44]. However, we found that RAF heterodimers were difficult to observe in trametinib-treated, acquired MEKi resistance cells (Figure 5E and Figure S6L). Importantly, cotargeting ABL1/2 and DDR1 using nilotinib or GNF-5+DDR-IN-1 dramatically increased heterodimerization in the presence of trametinib (Figure 5E,F and Figure S6L). Nilotinib also reduced coupling of NRAS to CRAF and ERK to BRAF in both cell lines, and in SK-MEL-2MR, nilotinib reduced NRAS/ARAF binding (Figure 5E,F and Figure S6L). Thus, reversal of MEKi resistance induced by targeting ABL1/2 and DDR1, results in heterodimerization and degradation of RAF proteins, and reduces coupling of RAF to upstream regulators and downstream targets.

### 3.9. Allosteric ABL1/2 Inhibitors Prevent SK-MEL-147MR Survival by Inhibiting MYC and ETS1 Expression and Blocking β-Catenin Nuclear Localization

**Figure 6:**
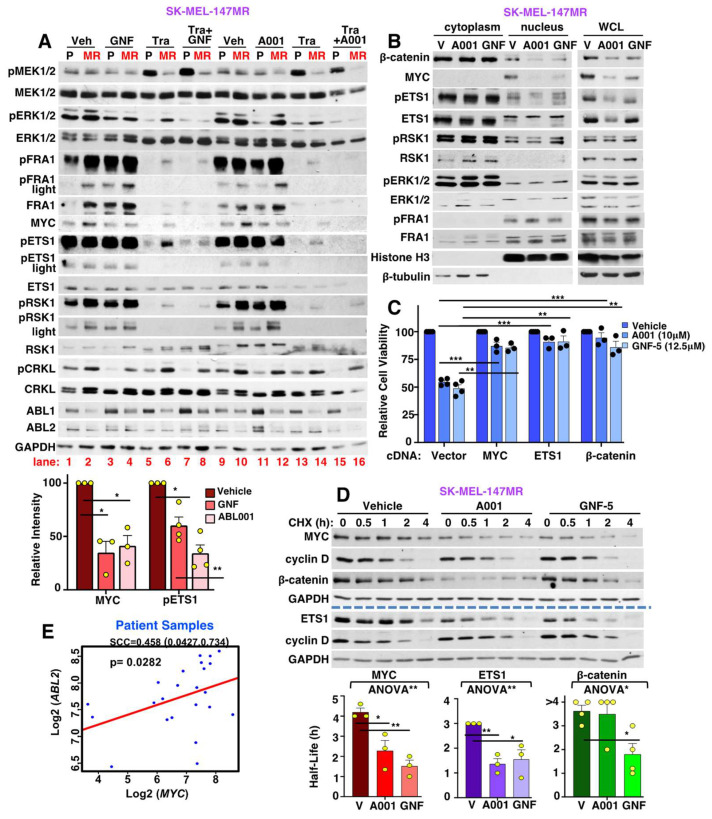
ABL1/2 drive trametinib resistance in SK-MEL-147MR by stabilizing MYC/ETS1 and inducing β-catenin nuclear localization. Lysates from cells treated with vehicle (Veh = V, DMSO), GNF-5 (12.5 μM), or ABL001 (A001, 10 μM), in the absence or presence of trametinib (Tra, 20 nM) for 48 h, were utilized for Western blotting (**A**) or subcellular fractionation (NE-PER; B). (**A**) Quantitation is mean ± SEM for n = 3. * *p* < 0.05, ** *p* ≤ 0.01. Exact *p*-values (left→right) = 0.042, 0.042, 0.015, 0.007. (**B**) Equal cytoplasmic protein was loaded, and relative equivalent fractions of nuclear lysates (1/2 volume of cytoplasmic fractions). Tubulin and Histone H3 show cytoplasmic and nuclear fraction purity, respectively. Representative blot for n=3 is shown. (**C**) Cells transfected with MYC, ETS1, β-catenin cDNAs, or vector were treated for 72 h with DMSO (Veh), GNF-5, or ABL001 (doses above) and viability was assessed with CellTiter Glo. Mean ± SEM for n = 3. * *p* < 0.05, ** *p* ≤ 0.01, *** *p* < 0.001. Holm’s adjusted *p*-values (left→right) = 0.000185, 0.001, 0.000103, 0.001, 0.000214, 0.001. (**D**) Protein stability assays. Cells were treated with GNF-5 or ABL001 for 48 h followed by cycloheximide (CHX; 100 μM) for the indicated times. Graphs are mean ± SEM for n = 3. Brackets indicate overall *p*-values for one-way ANOVAs: MYC: ** *p* = 0.0046, ETS1: ** *p* = 0.0078, β-catenin: * *p* = 0.0215. Bonferroni multiple comparison tests were used to compare treatment groups vs. DMSO. MYC: * *p* = 0.018, ** *p* = 0.0036; ETS1: ** *p* = 0.0082, * *p* = 0.0143; β-catenin: * *p* = 0.0247. (**E**) RNA-seq from patients with mutant *NRAS*-melanomas treated with a MEKi were analyzed with Spearman’s rank-order correlation. Correlation coefficients (r), 95% confidence limits, and *p*-values (2-tailed) are shown. The uncropped blots are shown in File S1.

SK-MEL-147MR are extremely sensitive to highly specific, allosteric ABL inhibitors (Figure 2G–I) in the absence of trametinib, even though they are less efficient than nilotinib at inhibiting ABL1/2 activity (pCRKL blots; Figure 6A vs. Figure 4A). The reduced efficiency of GNF-5 and ABL001 may be due to strong compensatory upregulation of ABL1 and/or ABL2 protein (Figure 6A). Interestingly, on their own, GNF-5 and ABL001 had little to no effect on pERK in resistant cells, but effectively inhibited activation/nuclear localization of the transcription factors ETS1, MYC, and β-catenin (Figure 6A,B, compare lane 4 to 2 and lane 12 to 10). Importantly, expression of exogenous MYC, ETS1, or β-catenin rescued GNF-5/ABL001-mediated reduction in survival (Figure 6C), indicating that all three transcription factors are required for ABL1/2-driven survival. MYC is transcriptionally regulated by ETS1 and β-catenin, in addition to ERK [7,45]. However, exogenous expression of ETS1 or β-catenin only partially rescued the inhibitory effects of ABL001/GNF-5 on MYC (Figure S7A), indicating that ABL1/2-mediated regulation of ETS1 and β-catenin is only partially responsible for increased MYC expression. Importantly, GNF-5/ABL001 also decreased the stability of MYC and ETS1, and GNF-5 destabilized β-catenin (Figure 6D). In contrast, neither drug affected cyclin D stability. Thus, ABL1/2 are critically important for the survival of SK-MEL-147MR cells due to their ability to induce ETS1/β-catenin-mediated induction of MYC and stabilize MYC and ETS1 proteins.

In contrast to their effects on their own, in the presence of trametinib, GNF-5 and ABL001 efficiently inhibited activation of ERK and downstream nuclear and cytoplasmic targets (FRA1/ETS1/RSK1/MYC) (Figure 6A, compare lane 8 to 6 and 16 to 14), similar to the effects of nilotinib (Figure 4A). Moreover, expression of constitutively active forms of ABL1/2 in SK-MEL-147P parental cells was sufficient to drive ERK pathway activation in the presence of trametinib, whereas silencing ABL1/2 in resistant SK-MEL-147MR cells reduced pERK/MYC/pETS1/pRSK1 in the presence of trametinib (Figure S7B,C). Thus, in SK-MEL-147MR cells, allosteric ABL inhibitors act on their own to prevent survival via ERK-independent effects on MYC, ETS1, and β-catenin, whereas ABL inhibitors reverse trametinib resistance via an ERK-dependent mechanism.

Since ABL1/2 activation of MYC is involved in trametinib resistance in all three resistant cell lines, we assessed whether ABL1/2 and MYC mRNA levels correlate in patient samples. We examined RNA-seq data in clinical trial samples from patients harboring *NRAS*-mutant melanomas who had been treated with a MEKi [46]. Interestingly, *ABL2* mRNA correlated with *MYC* mRNA expression in this dataset (Figure 6E), indicating that our data have clinical relevance.

### 3.10. Targeting ABL1/2 and DDR1 Reverses Intrinsic/Adaptive Resistance by Preventing Activation of Cytoplasmic but Not Nuclear ERK Targets

**Figure 7:**
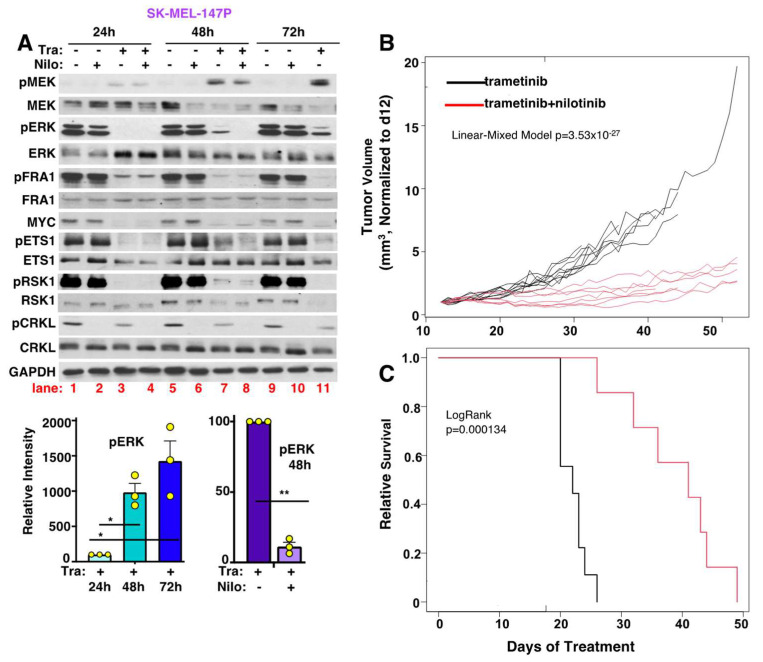
Targeting ABL1/2 and DDR1 blocks intrinsic/adaptive MEKi resistance, delays the onset of acquired resistance, and prolongs survival, in vivo. (**A**) Western blots from SK-MEL-147P parental cells treated with vehicle (DMSO, -), trametinib (Tra, 20 nM), nilotinib (Nilo, 2.5 μM), or the combination for the indicated times. Quantitation is mean ± SEM, n = 3. * *p* < 0.05, ** *p* ≤ 0.01. Exact *p* values (left→right) = 0.038, 0.042, 0.0012. (**B**,**C**) SK-MEL-147P parental xenografts were established in nude mice (≅200 mm^3^), followed by randomization to vehicle, nilotinib [33 mg/kg (d1–11); 50 mg/kg (d12–56), b.i.d.)], trametinib (1 mg/kg, once daily), or trametinib+nilotinib groups. Mice were treated with a lower dose of nilotinib (33 mg/kg) prior to elevating the dose (on d12) in order to reduce toxicity. (**B**) Individual log-transformed tumor growth curves (checked for linearity) for mice on d12–56 are normalized to d12 (baseline; division). Mean ± SEM tumor volume for all groups is shown in Figure S8A. Based on interaction between time and treatment, there is a significant difference between groups; *p* = 3.53 × 10^−27^ (Linear Mixed Model analysis). (**C**) Survival analysis using tumor doubling as the threshold. The legend is the same as in (**B**). LogRank *p* = 0.000134. trametinib, n = 9; trametinib+nilotinib, n = 7. Mouse weights are shown in Figure S8B. The uncropped blots are shown in File S1.

In addition to reversing acquired resistance, nilotinib also efficiently reversed partial intrinsic resistance in SK-MEL-147P parental cells (Figure 1F,G). Interestingly, although trametinib efficiently inhibited MEK/ERK activation at 24 h, MEK/ERK became reactivated at 48–72 h, indicating that the “intrinsic” resistance is actually short-term adaptive resistance (Figure 7A, compare lanes 3 and 7) [47]. Importantly, treatment with nilotinib efficiently blocked MEK/ERK reactivation at the 48 h timepoint (Figure 7A, lanes 7 and 8), and induced apoptosis (Figure 1H, 48 h timepoint is shown). Furthermore, at 72 h, combination-treated cells were completely dead and could not be blotted. Interestingly, MEK/ERK reactivation at the 48 h timepoint resulted in activation of cytoplasmic (ETS1, RSK1) but not nuclear (FRA1, MYC) ERK downstream targets, which are subsequently inhibited by the addition of nilotinib (Figure 7A). Thus, nilotinib reverses cytoplasmic and nuclear MEK/ERK signaling during acquired resistance but specifically suppresses cytoplasmic ERK signaling to reverse adaptive resistance.

### 3.11. Nilotinib Reverses Intrinsic MEKi Resistance, Delays the Onset of Acquired Resistance, and Prolongs Survival, In Vivo

In patients, MEKi are ineffective due to a combination of intrinsic/adaptive and acquired resistance. To mimic this situation, in vivo, we established SK-MEL-147 parental xenografts. Due to intrinsic/adaptive resistance, tumors were initially relatively resistant to trametinib alone, maintaining a stable size for 20–25 days at which time they acquired complete resistance, and their growth accelerated (Figure 7B and Figure S8A). In contrast, treatment upfront with nilotinib in combination with trametinib reversed intrinsic resistance, induced early tumor regression, delayed the onset of acquired resistance, and doubled the survival time (Figure 7B,C). Overall, animal weights were not significantly different between trametinib vs. combination-treated mice over the course of the experiment (e.g., *p* = 0.2 on d35; Figure S8B), suggesting that the combination is well-tolerated. In summary, these exciting data indicate that targeting ABL1/2 and DDR1 in combination with MEKi may be an effective therapeutic regimen for patients with aggressive *NRAS*-driven melanomas, who have a poor prognosis and limited therapeutic options.

## 4. Discussion

**Figure 8:**
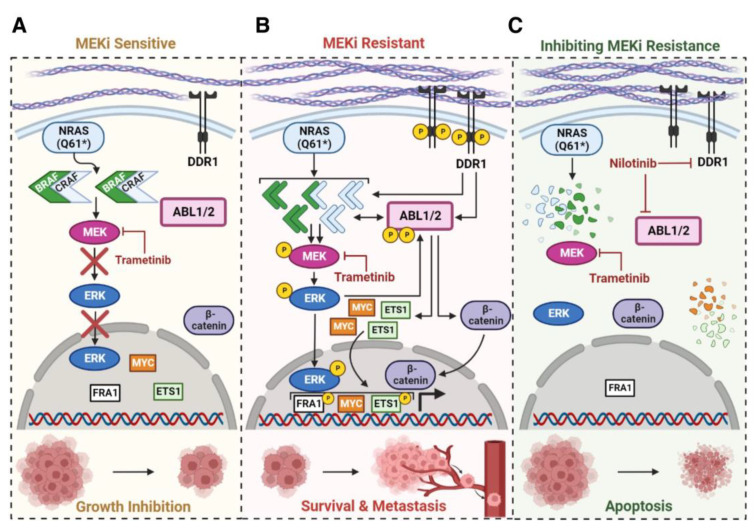
Summary diagram. (**A**) *NRAS*-mutant melanomas induce BRAF/CRAF dimerization and subsequent activation of MEK→ERK signaling. Treatment with MEKi (trametinib) inhibits MEK→ERK and causes tumor shrinkage. * indicates an activating mutation in NRAS (e.g., Q61K, Q61R, etc). (**B**) Prolonged treatment with MEKi leads to activation of ABL1/2 and DDR1 as a consequence of DDR1 binding increased collagen, and CRAF/ERK/DDR1 activating ABL1/2. Activation of ABL1/2 and DDR1 induces stabilization of BRAF and CRAF, and BRAF and CRAF homodimerization. BRAF and CRAF dimers are known to prevent trametinib from binding the allosteric binding site on MEK, which leads to MEK→ERK activation. ERK activation induces expression of FRA1, MYC, and ETS1 and ABL1/2 activation drives β-catenin nuclear localization and ETS1 and MYC1 stabilization. As a result, cells become resistant to MEKi, which is known to drive highly aggressive and metastatic disease. (**C**) Targeting ABL1/2 and DDR1 with nilotinib or GNF-5+DDR-IN-1 results in degradation of BRAF, CRAF, ETS1, and MYC, loss of MEK→ERK→FRA1 activation and nuclear localization of β-catenin, inhibition of proliferation, induction of apoptosis, and subsequent tumor regression. This diagram was created with Biorender (Biorender.com; URL accessed on 10 October 2022).

*NRAS*-mutant melanomas are a highly aggressive subtype and there is an unmet need to identify second-line treatment options for patients who fail to respond to immune-checkpoint blockade. In this manuscript, we describe a potential new therapy for patients with this subtype by characterizing the mechanism by which *NRAS*-mutant melanoma cells resist the effects of MEKi via short-term adaptive/intrinsic resistance and long-term acquired resistance. In some models, long-term acquired MEKi resistance was driven by activation of ABL1/2 and DDR1, which cooperated to drive resistance. This conclusion is based, in part, on loss-of-function studies in acquired resistant cell lines using siRNA and pharmacological approaches which demonstrated that ABL1/2 and DDR1 are both required to drive MEKi resistance. Moreover, we also utilized complementary gain-of-function studies to show that activation of DDR1, induced by plating parental cells on collagen I, together with expression of activated forms of ABL1/2, was sufficient to drive MEKi resistance. Furthermore, we identified the mechanism by demonstrating that ABL1/2 and DDR1 cooperated to activate ERK cytoplasmic and nuclear signaling (MYC/ETS1/RSK1), stabilize RAF and MYC proteins, and promote RAF homodimerization (Figure 8). In contrast, other long-term MEKi acquired resistance cells were highly dependent solely on ABL1/2, which stabilized MYC and ETS1 proteins in an ERK-independent manner, and induced β-catenin nuclear localization to drive melanoma survival (Figure 8). Finally, we showed that intrinsic/adaptive resistance occurs via a different mechanism as it is driven by reactivation of cytoplasmic but not nuclear ERK targets, which was reversed by the ABL1/2 and DDR1 inhibitor, nilotinib.

Interestingly, we found that CRAF and ERK as well as DDR1 contributed to ABL1/2 activation in *NRAS*-mutant cells that acquired long-term MEKi resistance. While receptor tyrosine kinases such as EGFR, PDGFR, and IGF1R have previously been implicated in ABL1/2 activation in other cell contexts [28,48,49,50], this is the first demonstration that DDR1 contributes to ABL1/2 activity. In contrast, DDR1 is likely activated by mRNA upregulation of collagen subunits in resistant cells. Indeed, plating parental cells on collagen activated DDR1 which cooperated with activated forms of ABL1/2 to drive resistance.

Using next generation sequencing, we identified new acquired mutations in GNAI1 (G125E; SK-MEL-30MR) and EPHA4 (N140fs, SK-MEL-2MR), RAF overexpression, and ABL1/2 and DDR1 as critical nodes that stabilized RAF and MYC proteins and destabilized p27/KIP1 while also upregulating MYC mRNA and repressing p27/KIP1 mRNA. ABL1/2 and DDR1 inhibition with either nilotinib or GNF-5+DDR-IN-1 (in the presence of trametinib) increased RAF heterodimerization in resistant lines, and reduced RAF binding to upstream regulators (NRAS), and downstream targets (ERK). Dimerization is required for RAS activation of wild-type RAF, and binding of 14-3-3 to RAF proteins not only retains RAF in an inactive conformation, but also is required for RAF dimerization and activation [51]. Heterodimerization is also enhanced by 14-3-3 proteins whereas ERK phosphorylation of BRAF-T753 promotes heterodimer disassembly [52]. ABL1 and DDR1 bind 14-3-3 proteins in other cell contexts [53,54], and here, we showed that ERK phosphorylation of ABL2 created a putative 14-3-3 binding site. Thus, ABL1/2 and DDR1 may impact heterodimerization and assembly of the NRAS/RAF/MEK/ERK complex by influencing RAF/14-3-3 scaffold interactions. Interestingly, peptides/drugs that bind the RAF dimerization motif not only prevent RAF dimerization but also induce RAF degradation [51], indicating that the effects of ABL1/2 and DDR1 inhibition on RAF heterodimerization and degradation may be linked.

Marais and colleagues demonstrated that some ABL1/2 and DDR1 inhibitors weakly bind RAF (following 3 h treatment), and induce RAF heterodimerization and paradoxical MEK/ERK activation in chronic myelogenous leukemia cells harboring a drug-resistant form of BCR-ABL (T315I) and tumor cells with activated RAS [55]. In contrast, while we showed that nilotinib treatment (24 h) induced RAF heterodimerization in MEKi-resistant *NRAS*-mutant melanoma cells, the result is inhibition of MEK/ERK and decreased coupling of NRAS and/or ERK to RAF rather than MEK/ERK activation. Moreover, since nilotinib’s effects are phenocopied by GNF-5+DDR-IN-1, the effects of nilotinib on RAF heterodimerization are likely not drug-dependent but rather are due to ABL1/2 and DDR1 inhibition.

Unlike BRAF and CRAF, silencing ARAF had little impact or even increased pERK activation (SK-MEL-30MR), and nilotinib increased ARAF mRNA expression in SK-MEL-30MR. ARAF was also absent from BRAF or CRAF complexes in SK-MEL-30MR cells unless nilotinib was present (Figure S6A, left). These data are consistent with published reports indicating a potential tumor suppressive role for ARAF in some cell contexts [56,57].

In contrast to the other two acquired MEKi-resistant cell lines, SK-MEL-147MR cells gained a MEK/ERK pathway mutation (MEK1; F129L). Although previously identified as an activating mutation that drives BRAFi resistance, this mutation was insufficient to drive MEK1/2 activation in SK-MEL-147MR. Phosphorylation of MEK1/2 by upstream RAF kinases and other MAP3Ks can reduce trametinib binding and cooperate with low transforming *MEK* mutations [42]. Indeed, we also identified a mutation in *MAP3K19*, a kinase that phosphorylates MAP2Ks including MEK1/2 in SK-MEL-147MR cells (Table S2 and Dataset S2) [58].

Our exciting in vivo data have uncovered a potential new treatment for NRAS-driven melanomas, an understudied and highly aggressive subtype with notoriously poor outcomes. ABL1/2 and DDR1 inhibition in combination with MEKi not only reversed established and intrinsic resistance in vivo, but also significantly delayed acquired resistance from developing in the first place, and substantially increased survival. Since a number of ABL1/2 and DDR1 inhibitors are FDA-approved for treating leukemia, these data may pave the way for testing their clinical efficacy in combination with MEK for treatment-refractory NRAS-driven melanomas.

## 5. Conclusions

*NRAS*-mutant metastatic melanoma is an aggressive and deadly disease with no effective second-line treatments. In this manuscript, we demonstrated that in some resistant lines, ABL1/2 cooperate with DDR1 to drive MEKi acquired resistance by inducing activation of ERK/MYC/ETS1/RSK1, stabilizing RAF and MYC, and promoting RAF homodimerization (Figure 8). In other resistant lines, MEKi-resistant cells were highly dependent solely on ABL1/2, which promoted survival by stabilizing MYC and ETS1, in an ERK-independent manner, and inducing β-catenin nuclear translocation (Figure 8). Finally, ABL1/2 and DDR1 are also critical nodes during intrinsic resistance and are required for adaptive reactivation of cytoplasmic ERK targets. Significantly, using a mouse model that mimics intrinsic and acquired MEKi resistance, targeting ABL1/2 and DDR1, with an FDA-approved drug, reversed intrinsic resistance and delayed the onset of acquired MEKi resistance in vivo, thereby enhancing survival. Thus, repurposing inhibitors targeting ABL1/2 and DDR1, many of which are FDA-approved, may be an effective therapeutic approach, in combination with MEKi, for patients with metastatic *NRAS*-mutant melanomas that fail to respond to immune-checkpoint blockade and thus, have no effective second-line treatment options.

## Data Availability

Next generation sequencing data (whole exome and RNA-seq) generated in this manuscript are included as Supplementary Material. The raw files have been deposited in the NCBI/SRA database (Bioproject Accession number PRJNA887367, see Table S1).

## Supplementary Materials

The following supporting information can be downloaded at: https://www.mdpi.com/article/10.3390/cancers15030954/s1. Figure S1. ABL1/2 and DDR1 cooperate to drive trametinib resistance. Figure S2. Geneset pathway changes in resistant lines. Figure S3. ERK phosphorylates ABL1 and ABL2. Figure S4. ABL1/2 and DDR1 promote ERK pathway reactivation. Figure S5. Geneset pathway changes in resistant lines treated with trametinib+nilotinib as compared to trametinib alone using RNA sequencing. Figure S6. Nilotinib reduces BRAF/CRAF stability and alters RAF dimerization. Figure S7. ABL1/2 are necessary and sufficient to drive survival and resistance in SK-MEL-147 cells. Figure S8. Nilotinib prevents resistance in vivo. Table S1. Reagents table. Table S2. Next generation whole exome sequencing. Table S3. RNA sequencing of trametinib vs. trametinib+nilotinib-treated resistant cell lines. Dataset S1. RNA sequencing comparing parental and resistant cell lines. Dataset S2. Whole exome sequencing comparing resistant to parental cell lines. Dataset S3. Gene changes for RNA sequencing for trametinib vs. trametinib+nilotinib-treated resistant cells. Supplementary Methods and References. References [59,60,61,62,63,64,65,66,67,68,69,70,71,72,73] are cited in the Supplementary Materials section. File S1: Original western blots.
