# Supplementary material for: ABL1/2 and DDR1 Drive MEKi Resistance in NRAS-Mutant Melanomas by Stabilizing RAF/MYC/ETS1 and Promoting RAF Homodimerization
Source: Cancers (Basel). 2023 Feb 2;15(3):954. doi: 10.3390/cancers15030954 (PMC9913232; doi:10.3390/cancers15030954)
Supplement: Supplementary file 1 [file cancers-15-00954-s001.zip › cancers-1993363-supplementary/cancers-1993363-supplementary.pdf]

SUPPLEMENTARY MATERIALS

ABL1/2 and DDR1 drive MEKi resistance in NRAS-mutant melanomas by stabilizing RAF/MYC/ETS1 and promoting RAF homodimerization

Anastasia Lyon, Rakshamani Tripathi, Christina Meeks, Daheng He, Yuanyuan Wu, Jinpeng Liu, Chi Wang, Jing Chen, Haining Zhu, Sujata Mukherjee, Saptadwipa Ganguly, and Rina Plattner

This PDF file includes:

Supplementary Figures 1-8

Supplementary Tables 1-3

Legends for Supplementary Dataset Files 1-3

Supplementary Methods

Supplementary Methods References

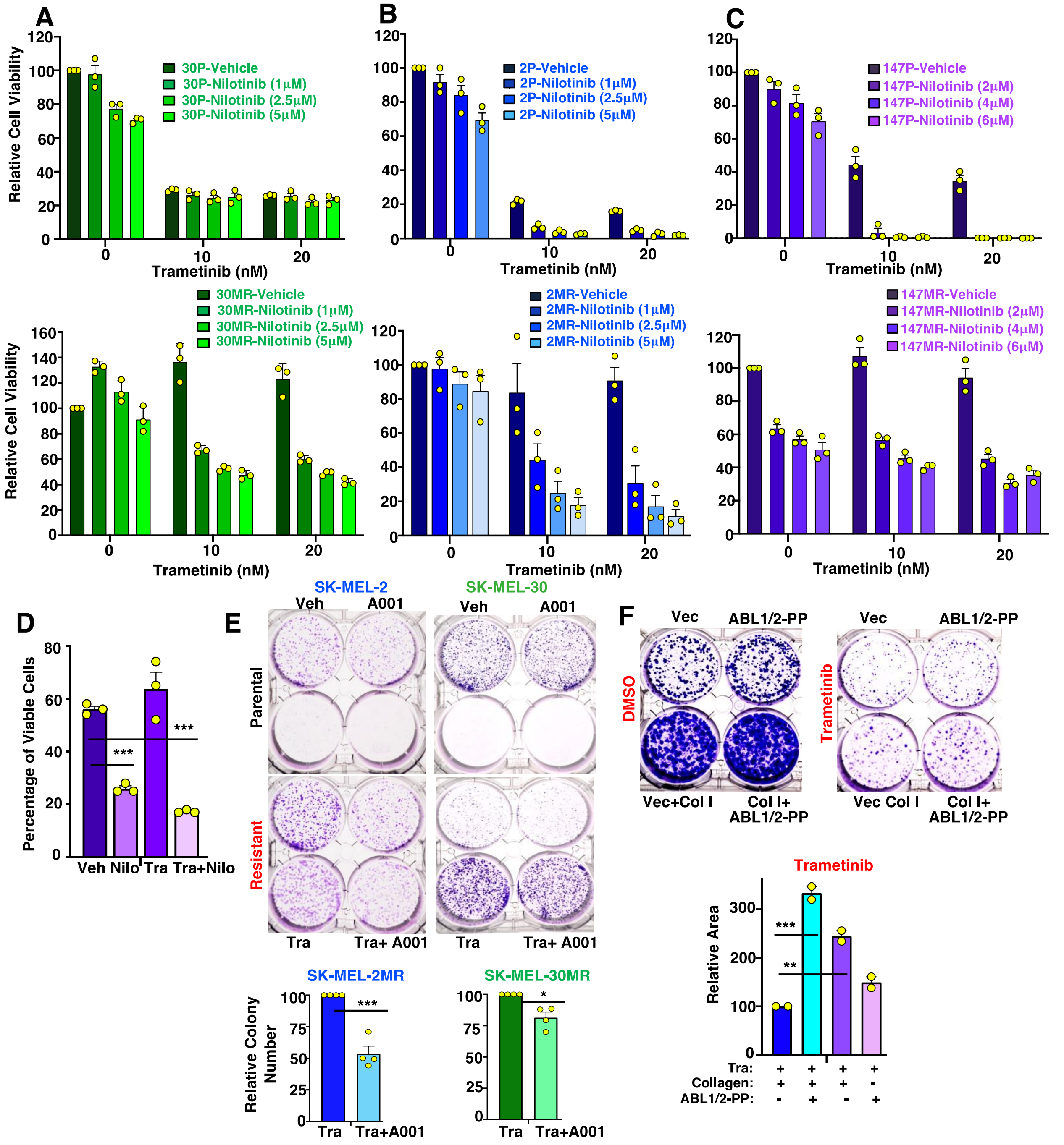

Supplementary Figure 1. ABL1/2 and DDR1 cooperate to drive trametinib resistance. (A-C) Cell viability (CellTiter Glo) assays. Parental (P) and resistant (MR) cell lines, plated in 96-well dishes in triplicate, were drug-treated for 72h (SK-MEL-30, SK-MEL-2) or 96h (SK-MEL-147) and harvested for CellTiter Glo viability assays. Mean±SEM for n=3. These figures support Fig. 1D-F (additional doses). **(D)** Cells were treated as in A-C for 96h, and live cells counted using trypan blue exclusion and TC-20 (Biorad) cell counter. Mean±SEM for n=3. One-way ANOVA p<0.0001. Bonferonni multiple comparison's test p values (left->right): 0.0006, <0.0001. (**E**) Cells were treated with ABL001 (10µM), -/+ trametinib (Tra, 20nM) for 7d, and colonies stained with crystal violet. ***p<0.001; *p=0.025. **(F)** Cells transfected with vectors or constitutively active ABL1/2 (PP, Fig. 2F) were plated on collagen I-coated or normal plates, drug-treated (1.5nM, 5-7d), and colonies stained 4d later. Mean±SEM for n=2. One-Way ANOVA p=0.0003; Bonferonni posthoc p-values: ***p=0.0003, **p=0.018.

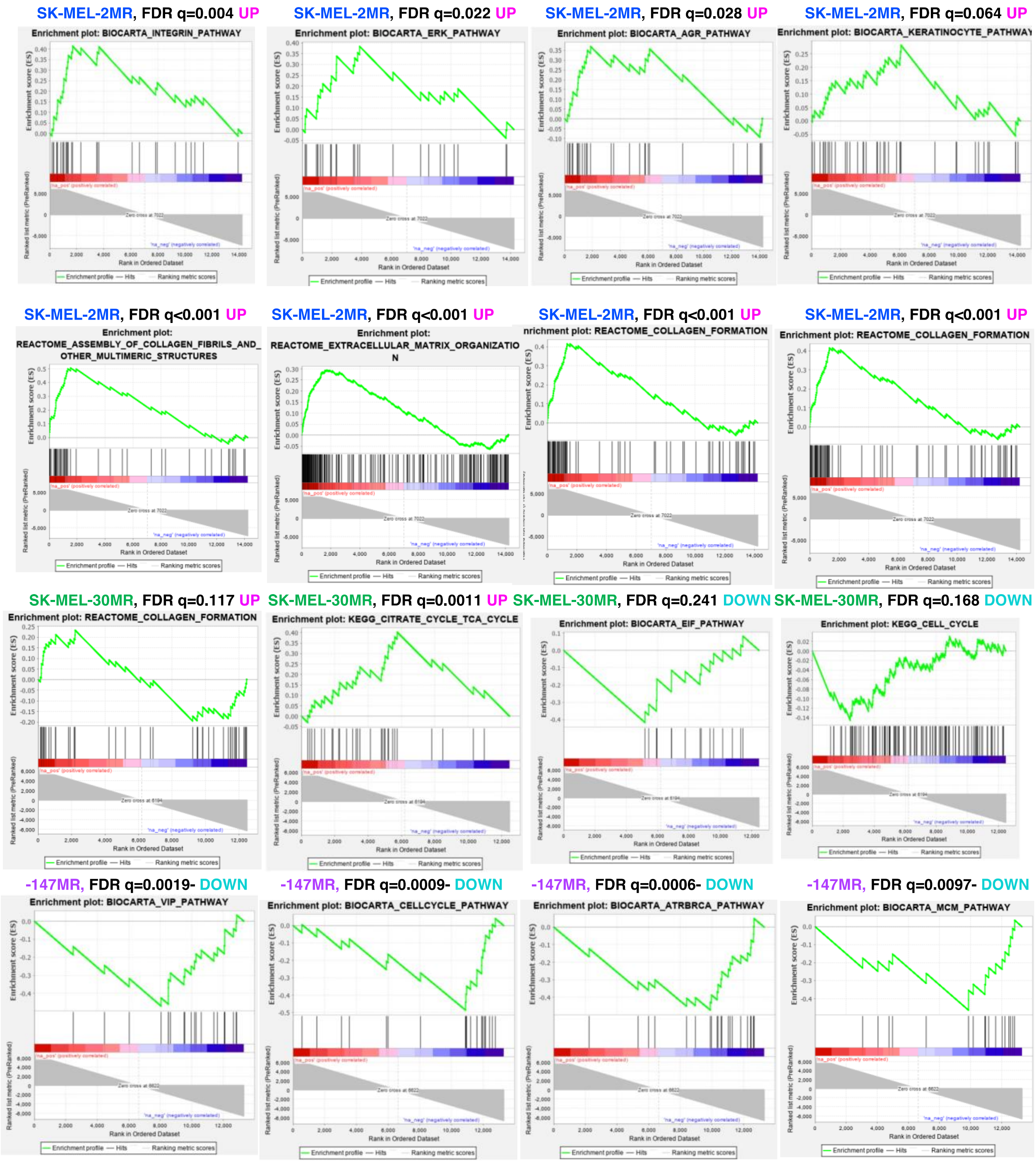

Supplementary Figure 2. Geneset pathway changes in resistant lines. RNA sequencing followed by Gene Set Enrichment Analysis (GSEA) comparing parental to resistant lines. Enrichment plots for Biocarta Pathways. UPregulation indicates geneset is increased in resistant line, whereas DOWNregulation indicates the reverse.

A

B

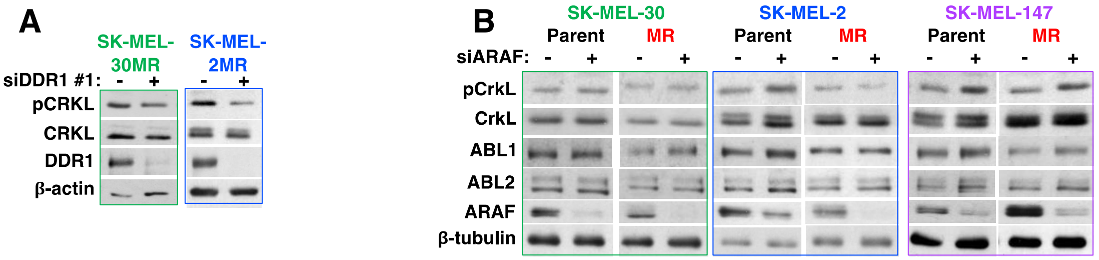

C

Substrate	Peptide	Amino Acid	Fold Change (Phos/Non-Phos)	Comment
ABL1b	GSALGTPAAAEPVTPTSK	T833	82	ERK consensus phosphorylation site
ABL1b	NEEAADEVFKDIMESSPGSSPPNLTPKPLRR	T871	3.5	
ABL2	GATALPLR	T843	No phosph in Con	Putative 14-3-3 binding site
ABL2	VPVLISPTK	S922	122	
ABL2	STQASSGSPALPR$_{656}$	T605	3.3	
ABL2	AGKPTASDDTSKPFPR	T759/S760	5.6	

D

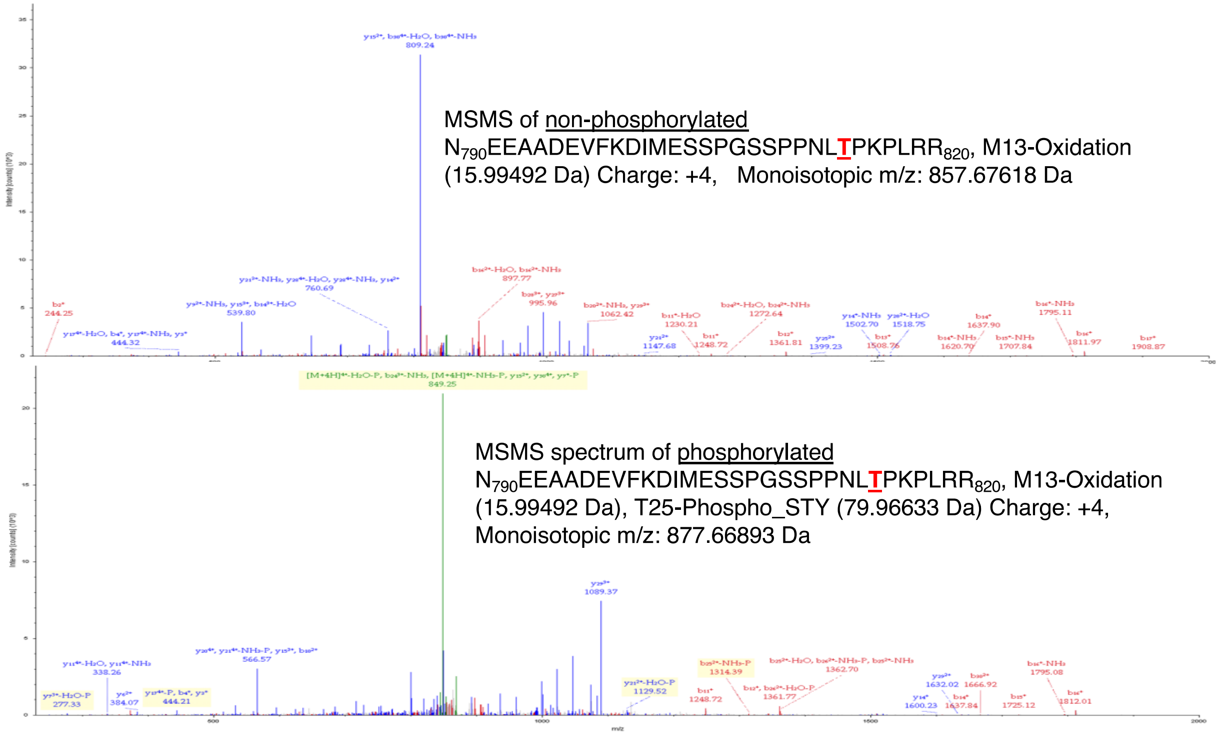

Supplementary Figure 3. ERK phosphorylates ABL1 and ABL2. (A) Western blots from cells transfected with DDR1 siRNA#1, assessing CRKL phosphorylation (pY207) a read-out of ABL1/2 activities. **(B)** Cells were transfected with ARAF siRNA (5nM) and lysates blotted after 72h. Some control blots are the same as in Fig. 5D as they come from the same replicate. **(C)** ERK-mediated phosphorylation sites on ABL1 and ABL2 identified by mass spectrometry. **(D)** Mass Spectra for NLTP phosphorylation site on ABL1. Top-non-phosphorylated peptide. Bottom-phosphorylated peptide.

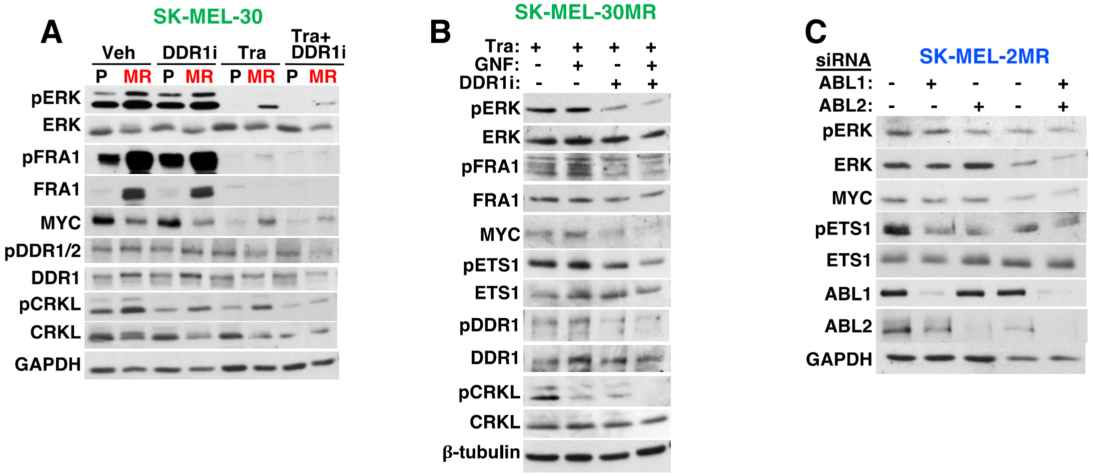

Supplementary Figure 4. ABL1/2 and DDR1 promote ERK pathway reactivation. (A,B) Western blots using lysates from cells treated for 24h (A) or 48h (B). Trametinib (Tra)= 20nM, DDR-IN-1 (DDR1i)=4μM (A), 2.5μM (B); GNF-5=12.5μM. **(C)** Western blot analysis using trametinib-resistant cell lines, transfected with ABL1 and/or ABL2 siRNAs (20nM) for 72h. pCRKL/CRKL blots in (A) are from the same experiment shown in Fig. 3A.

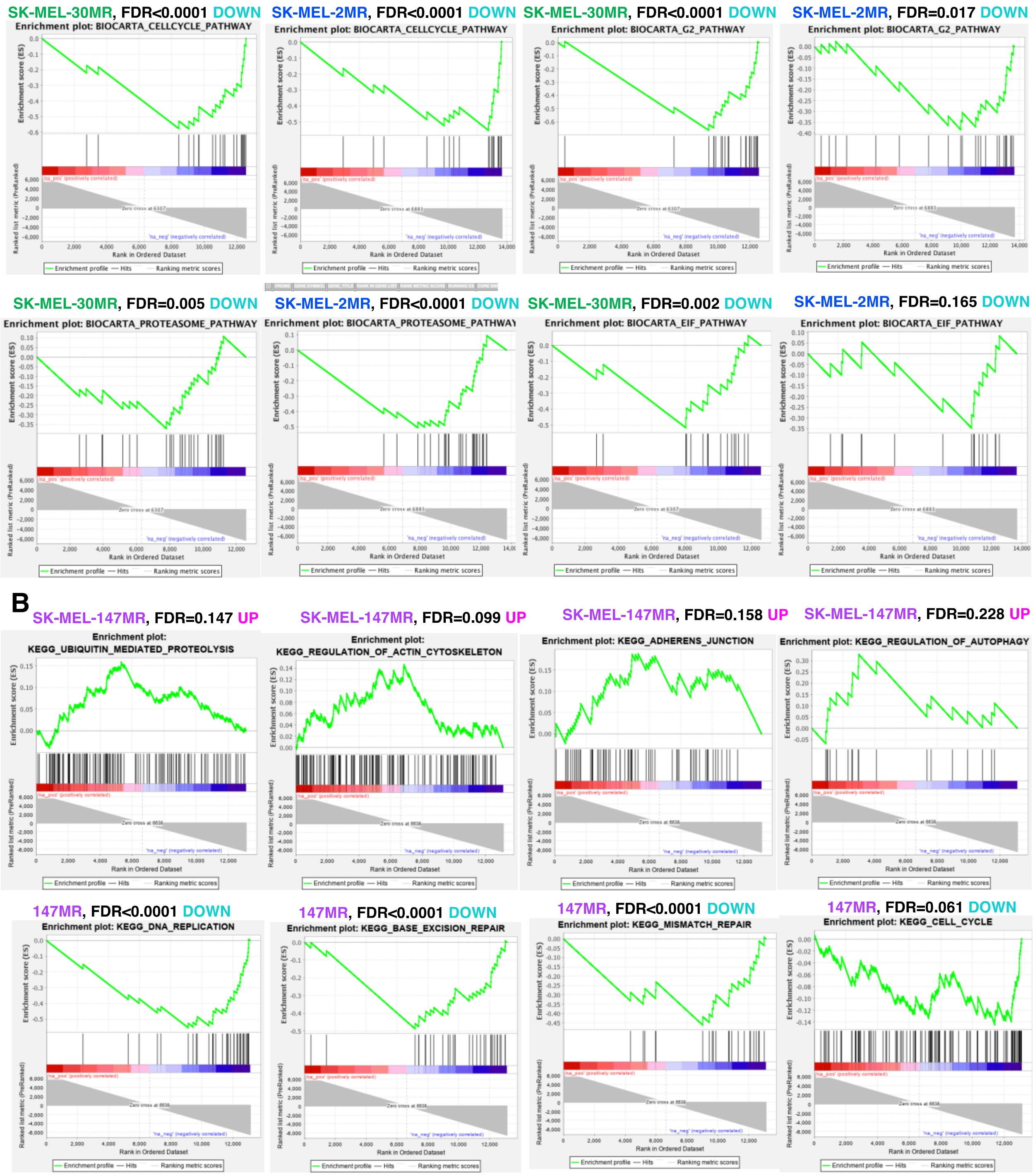

Supplementary Figure 5. Geneset pathway changes in resistant lines treated with trametinib+nilotinib as compared to trametinib alone using RNA sequencing. UPregulation indicates geneset is increased in trametinib+nilotinib-treated cells, whereas DOWNregulation indicates the reverse. Biocarta pathways are shown. **(A)** SK-MEL-2MR and SK-MEL-30MR; **(B)** SK-MEL-147MR

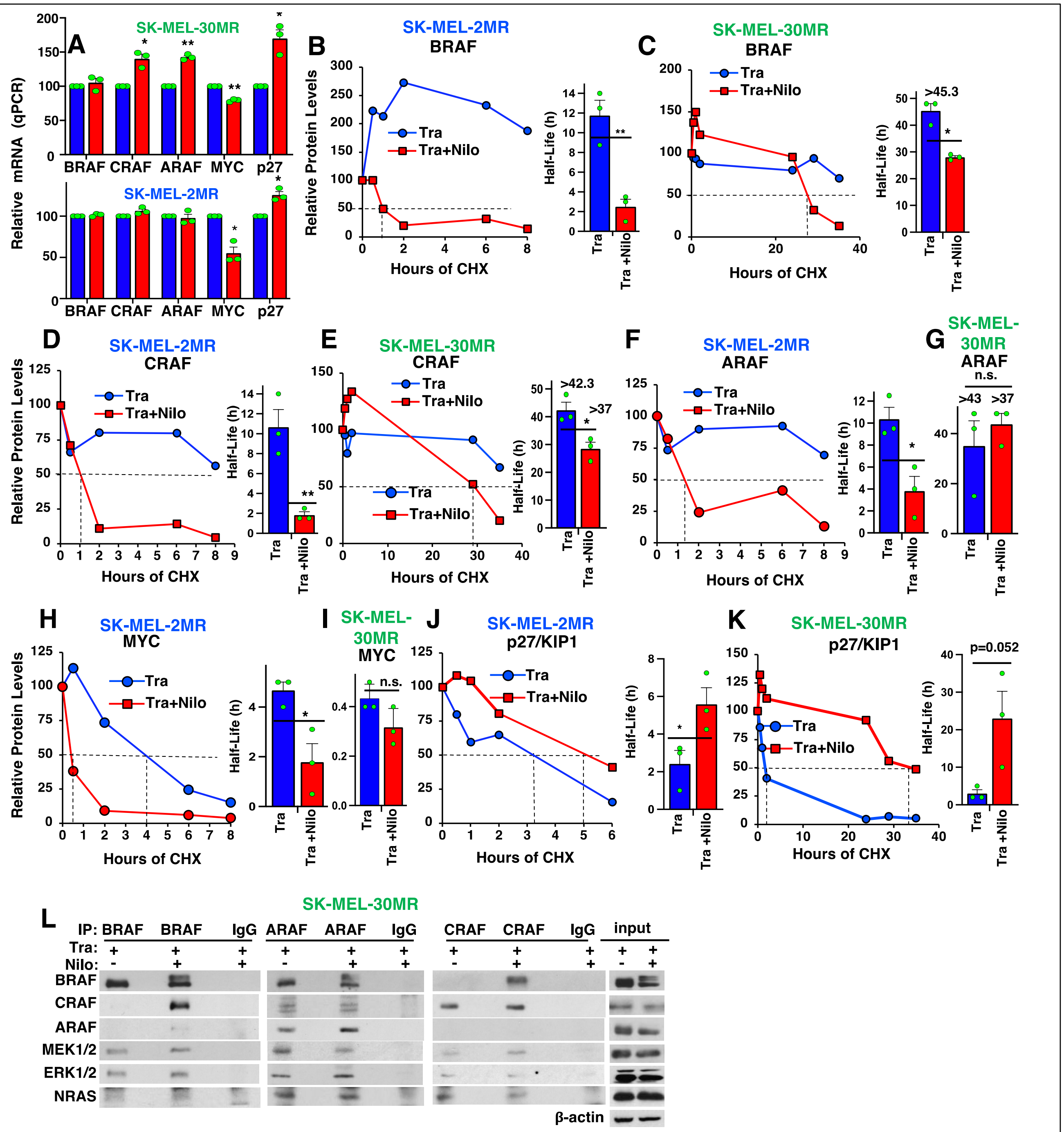

Supplementary Figure 6. Nilotinib reduces BRAF/CRAF stability and alters RAF dimerization. **(A)** qPCR using RNA from cells treated with trametinib or trametinib+nilotinib for 24h. Mean±SEM for n=3. *p<0.05, **p≤0.01,***p<0.001. Exact *p*-values: SK-MEL-30MR, CRAF=0.027, ARAF=0.003, MYC=0.0034, p27/KIP=0.031; SK-MEL-2MR, MYC=0.027, p27/KIP=0.027. **(B-K)** Quantitation of CHX assays shown in Fig. 5B. Black dotted lines indicate 50% loss. Bar graphs represent Mean±SEM from n=3. Half-lives that are >8h for SK-MEL-2MR were estimated with Excel's linear forceast. For SK-MEL-30MR, half-lives greater than 48 were denoted >48h. *p*-values [**B:** 0.006; **C:** 0.0031; **D:** 0.0079; **E:** 0.02; **F:** 0.018; **H:** 0.024; **J:** 0.049; **K:** 0.052. (L) Coimmunoprecipitation studies. IPs from treated lysates (24h) were blotted (2nd line supporting Fig. 5E).

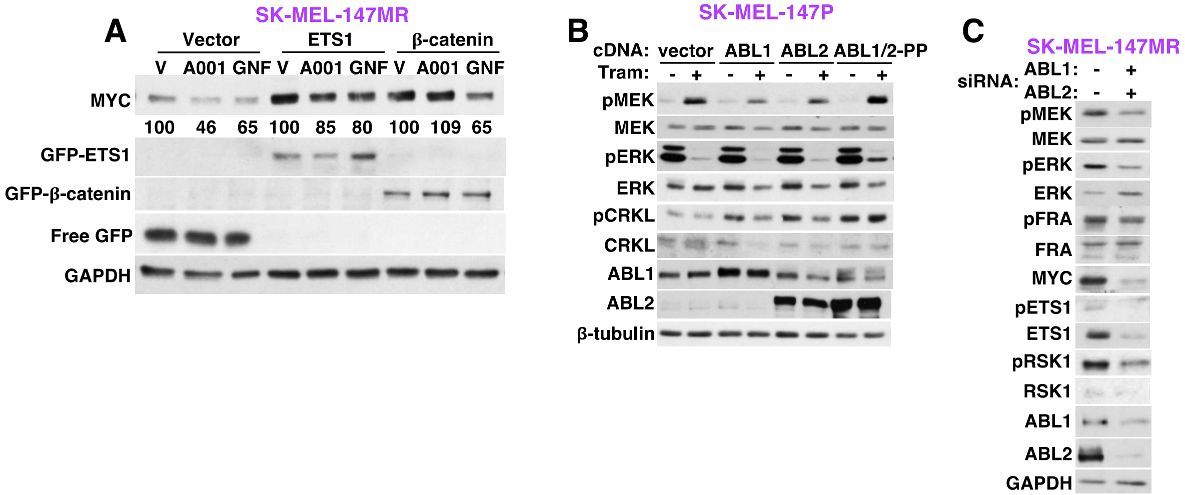

Supplementary Figure 7. ABL1/2 are necessary and sufficient to drive survival and resistance in SK-MEL-147 cells. **(A)** Cells transfected with ETS1 or beta-catenin plasmids (or vector) were treated with vehicle, GNF-5 (12.5μM) or ABL001 (A001; 10μM) for 48h, and lysates blotted. A representative of n=3 is shown. **(B)** Western blots of lysates from parental cells engineered to express cumate-inducible, constitutively active ABL1, ABL2 or ABL1+ABL2 (PP). Cells were treated with cumate (0.5X; 24h) -/+ trametinib (10nM). **(C)** Western blots using lysates from cells transfected with ABL1 and ABL2 siRNAs followed by trametinib treatment for 24h.

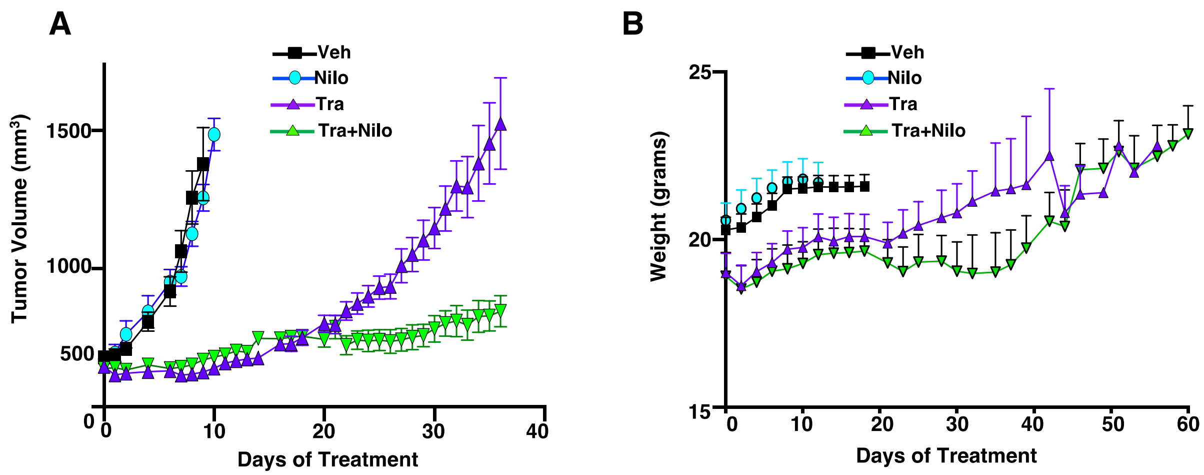

Supplementary Figure 8 . Nilotinib prevents resistance, *in vivo*. **(A,B)** Xenograft experiment from Fig. 7B,C. Mean±SEM tumor volumes **(A)** and animal weights **(B).** Note, animals began to be lost from the trametinib group (euthanized due to tumor size or ulceration) on d30. **(A)** Means are shown for days in which ≤3 animals/group are lost. Vehicle, n=12; nilotinib (Nilo), n=12; trametinib (Tra), n=9; trametinib+nilotinib (Tra+Nilo), n=7.

SUPPLEMENTARY TABLES

Table S1. Reagents Table

REAGENT or RESOURCE	SOURCE	IDENTIFIER
Antibodies		
ABL1	Santa Cruz Biotech (Santa Cruz, CA)	cat#56887; clone 8E9; RRID:AB_781732
ABL2	Millipore Sigma (St. Louis, MO)	5C6; RRID:AB_1839297
AKT	Cell Signaling (Danvers, MA)	9272; RRID:AB_329827
AKT (pS473)	Cell Signaling	4060; RRID:AB_2315049
ARAF	Santa Cruz Biotech	cat#166771; 16671-AC; clone A-5; RRID:AB_2060508
Beta-actin	Millipore Sigma	cat# A5316; Clone AC-74; RRID:AB_2861213
Beta-catenin	Santa Cruz	Cat#7963; clone E-5'RRID:AB_626807
Beta-tubulin	Santa Cruz Biotech	Cat#166729; clone F-1; RRID:AB_ 2010699
BRAF	Santa Cruz	Cat#5284, 5284-AC; clone F-7; RRID:AB_626760
Cleaved caspase-3	Cell Signaling	Cat#9664; RRID:AB_2070042
Cleaved PARP	Cell Signaling	Cat#5625; RRID:AB_10699459
CRAF	Santa Cruz	Cat#7267; 7267-AC; clone E-10; RRID:AB_628196
CRAF (pS338)	Cell Signaling	Cat#9427
CRKL	Cell Signaling	Cat#3182; RRID:AB_10693644
CRKL (pY207)	Cell Signaling	Cat#3181
Cyclin D	Millipore-Sigma	Cat#06-137; RRID:AB_11214218
DDR1	Santa Cruz Biotech	Cat#sc-532, clone C-20; RRID:AB_ 2092090
DDR1/2 (p796/pY740)	R&D (Minneapolis, MN)	Cat#25382
DDR1 (p792)	Cell Signaling	Cat#11994 RRID:AB_2797793
ERK	BD Biosciences (Franklin Lakes, NJ)	Cat#610123; RRID:AB_397529
ERK (pT202/pY204)	Promega/VWR (Atlanta, GA)	Cat#RB9249P; RRID:AB_430866
ETS1 (pT38)	Bioworld (Nanjing, China)	Cat#BS4316
ETS1	Santa Cruz Biotech	Cat#55581, clone C-4; RRID:AB_831289

FRA1	R&D	Cat#AF4935; RRID:AB_2107059
FRA1 (pS265)	Cell Signaling	Cat#3880; RRID:AB_2106922
GAPDH	ThermoFisher	AM4300; RRID:AB_2536381
GFP	Santa Cruz	Cat#9996 RRID: AB_627695
Histone H3	Cell Signaling	Cat# 9715S RRID:AB_331563
LC3B	Cell Signaling	Cat# 3868S; RRID:AB_2137707
MEK1/2	Cell Signaling	Cat#8727; RRID:AB_10829473
MEK1/2 (pS217/pS221)	Cell Signaling	Cat#9154; RRID:AB_2138017
MYC	R&D	Cat#MAB3696
NRAS	Santa Cruz	Cat#sc-31; RRID:AB_628041
p27	Cell Signaling	Cat#3688; RRID:AB_2077836
RSK (pS380)	R&D	Cat#MAP79671
RSK	Santa Cruz	Cat#393147
Anti-mouse HRP	ThermoFisher (Florence, KY)	Cat#PI31430; RRID:AB_228307
Anti-rabbit HRP	Jackson ImmunoResearch (West Grove, PA)	Cat#711-035-052
Anti-rabbit HRP	GE Healthcare/VWR	Cat#NA934V
Anti-goat HRP	Jackson ImmunoResearch	Cat#805-035-180
Normal mouse IgG-AC	Santa Cruz	Cat#2343
Chemicals, peptides, and recombinant proteins		
Nilotinib 2nd generation ABL/DDR1b inhibitor	Novartis (Basel, Switzerland)	MTA
GNF-5 ABL inhibitor	Selleck (Houston, TX)	Cat#S7526
ABL001 ABL inhibitor	Novartis	MTA
Trametinib MEK inhibitor	MedChemExpress (Monmouth Jnct, NJ)	Cat#HY10999
DDR-IN-1 DDR1 inhibitor	MedChemExpress	Cat#13979
PLX3397 KIT inhibitor	MedChemExpress	Cat#16749
CGP673451 PDGFR inhibitor	MedChemExpress	Cat#12050
SCH772984 ERK inhibitor	MedChemExpress	Cat#HY50846
Ponatinib 3rd generation ABL/DDR1 inhibitor	MedChemExpress	Cat#12047
Cumate for inducible system	Systems Bioscience (Palo Alto, CA)	Cat#QM100A
Cycloheximide	Millipore Sigma	Cat#C7698-1G
ECL	Pierce/ThermoFisher	Cat#PI32106
Recombinant GST-ERK2	ThermoFisher	Cat#3595; Jain et al. 2017

Recombinant His-ABL1	ThermoFisher	Cat#3049; Jain et al. 2017
Recombinant His-ABL2	ThermoFisher	Cat#PV3266; Jain et al. 2017
0.05% Crystal Violet Stain	Acros (Cole-Parmer) (Vermon Hills, IL)	Cat# EW-88226-98
methanol	VWR	Cat#BDH1135-19L
nitrocellulose	VWR	Cat#10063-173
High concentration matrigel	Corning Life Sciences/VWR	Cat#47747-220
Commercial Kits		
CellTiter Glo	Promega/VWR	Cat#PAG7572
Ambion Turbo DNase DNA-free kit	Ambion/ThermoFisher	Cat#AM1907
SYBR green	BioRad	Cat#1708890
iScript	BioRad	Cat#1725270
RNAeasy	Qiagen	Cat#74104
Deposited data		
Whole Exome Sequencing MR vs. Parental cell lines, Raw and Analyzed data	This paper	SRA BioProject: PRJNA887367<br>Sample Accession #s:<br>SRR21813786<br>SRR21813785<br>SRR21813784<br>SRR21813783<br>SRR21813798<br>SRR21813797
RNA-seq MR vs. Parental cell lines	This paper	SRA BioProject: PRJNA887367<br>Sample Accession #s:<br>SRR21813800<br>SRR21813799<br>SRR21813790<br>SRR21813789<br>SRR21813788<br>SRR21813787
RNA-seq MR trametinib vs. trametinib+nilotinib	This paper	SRA PRJNA887367<br>Sample Accession #s:<br>SRR21813796<br>SRR21813795<br>SRR21813794<br>SRR21813793<br>SRR21813792<br>SRR21813791
Reanalyzed data		
RNA-seq from *NRAS*-mutant melanoma patients on MEKi	Yardena Samuels/Mitchell Levesque	Nagler et al. 2020
Experimental models: Cell lines		
SK-MEL-2	NCI (Bethesda, MD)	NCI-60 panel, 2016
SK-MEL-2MR	This manuscript	

SK-MEL-30	Dr.Taha Merghoub, MSKCC<br>(New York, NY)	Via an MTA, 2018, 2022
SK-MEL-30MR	This manuscript	
SK-MEL-147	Dr.Taha Merghoub, MSKCC<br>(New York, NY)	Via an MTA, 2018, 2022
SK-MEL-147MR	This manuscript	
Experimental models: Organisms/strains		
Athymic nude mice (Nu/J)	Jackson Labs	Stock#002019
Oligonucleotides		
Scrambled (Silencer Select)	Ambion/ThermoFisher	Cat#4390844
Scrambled (Silencer)	Ambion/ThermoFisher	Cat#AM4635
siARAF	Ambion/ThermoFisher	Assay ID# s576
siBRAF	Ambion/ThermoFisher	Assay ID# S2080
siCRAF (RAF1)	Ambion/ThermoFisher	Assay ID# 11750
siDDR1	Ambion/ThermoFisher	Assay ID# S2298, S2299
siDDR1-3'UTR	Horizon<br>(Rockville, IL)	J-003111-12-0005
siABL1	Ambion/ThermoFisher	Assay ID# 1336,866
siABL2	Ambion/ThermoFisher	Assay ID# 1478,872
qPCR forward primer BRAF	Integrated DNA Technologies (IDT)	5'CTCCTTGAATCGGGC TGGTT3'
qPCR reverse primer BRAF	IDT	5'AGGAAACGCACCATA TCCCC3'
qPCR forward primer CRAF (RAF1)	IDT	5'TTAGGCCTCGTGGAC AGAGA3'
qPCR reverse primer CRAF (RAF1)	IDT	5'AAAGAGCCTGACCCA ATCCG3'
qPCR forward primer ARAF	IDT	5'ATTGTTCCTCCAAGG TCCCC3'
qPCR reverse primer ARAF	IDT	5'GTGGTAGAACCTTGA GGGCTG3'
qPCR forward primer MYC	IDT	5'CGGGTAGTGGAAAAC CAGCCT3'
qPCR reverse primer MYC	IDT	5'AGAAATACGGCTGCA CCGAG3'
qPCR forward primer p27/KIP1 (CDKN1B)	IDT	5'TCTGAGGACACGCAT TTGGT3'
qPCR reverse primer p27/KIP1 (CDKN1B)	IDT	5'AAGAATCGTCGGTTG CAGGT3'
qPCR forward primer RPS13 (reference gene)	IDT	5'CGAAAGCATCTTGAG AGGAACA3' [62]
qPCR reverse primer RPS13 (reference gene)	IDT	5'GCACCACGTCCAATA CAT3' [62]
Recombinant DNA		
PiggyBac-ABL1-PP	Barila et al. 1988 [25]<br>Jain et al. 2017 [22]<br>Tripathi et al. 2020 [59]	

PiggyBac-ABL2-PP	Barila et al. 1988 [25]<br>Jain et al. 2017 [22]<br>Tripathi et al. 2020 [59]	
MSCV-hMYC-IRES-GFP	Addgene (Watertown, MA)	Cat#18119; depositor, John Cleveland
pCMV-ETS1-IRES-eGFP	GeneCopoeia (Rockville, MD)	Cat#EX-Z1710-M61; clone MGC:29755
pEGFP-C1-beta-catenin	Addgene	Cat#16838; depositor, Erin Schuman
Software and algorithms		
Trimmomatic (V0.39)	Bolger et al. (2014) [60]	
BWA (V.0.7.17)	Li et al. (2010)	
Picard (v2.20.2)		http://broadinstitute.github.io/picard/
Genome Analysis Toolkit (GATK v4.1.2.0)	McKenna et al. (2010) [61]	
Mutect2	Cibulskis et al. (2013) [62]	
Oncotator (v.1.9.9.0)	Ramos et al. (2015) [63]	
RSEM (v1.3.2)	Li et al. (2011) [64]	
EdgeR	Robinson et al. (2010) [65]	
GSEA (v4.0.3)	Subramanian et al. (2005) [66]	
SAS (v9.4)		www.sas.com
R (v4.0.0)		https://cran.r-project.org
Proteome Discoverer (v1.3)	ThermoFisher	
ImageJ	NIH	https://imagej.nih.gov/ij/
Image J plugin ColonyArea	EUDAT CDI	https://b2share.eudat.eu/records/39fa39965b314f658e4a198a78d7f6b5
GraphPad Prism	GraphPad	https://www.graphpad.com
14-3-3 Pred (predicts 14-3-3 binding sites	Medeira et al. (2015) [30]	https://www.compbio.dundee.ac.uk/1433pred
Primer Blast	NCBI	https://www.ncbi.nlm.nih.gov/tools/primer-blast/
Biorender		https://biorender.com
Cell Culture Reagents		
Fetal Bovine Serum	Gibco/ThermoFisher	10437028
DMEM	Corning/VWR	45000-304
RPMI	Corning/VWR	45000-396
Glutamine	Corning/VWR	45000-676
Trypsin-EDTA	Corning/VWR	45000-660
Matrigel for animal injection	BD/Fisher	354248

HEPES	Fisher	Cat#11344-041
Lipofectamine 2000	Invitrogen/ ThermoFisher	Cat#11668-027

Resistant Cell Lines	MAPK Pathway Mutations
SK-MEL-30MR	GNAI1-G125E
SK-MEL-2MR	EPHA4-N140fs
SK-MEL-147MR	MAP2K1-F129L, MAP3K19-L946F

Table S2. Next Generation Whole Exome Sequencing. MAPK pathway acquired mutations in resistant lines that are not present in parental lines. See Dataset 2 for complete gene lists.

Resistant Cell Lines	Downregulated Tra/Nilo	Upregulated in Tra/Nilo
SK-MEL-30MR	FOS, FOSL1, DUSP4/6, ETV1/4/5, EPHA2, EGR1, SPRY1/2/4, CCND1,CDKN2D (Ink4d), CDC25A, MAFF, HMGA2, KCNN4, UBALD2, NEDD9, E2F2, E2F8, MAPK10, ERBB3 , MAP3K4	DUSP2, DUSP8, PRUNE2,
SK-MEL-2MR	FOSL1, DUSP4/5/6, ETV1/4/5, EPHA2, EGR1, SPRY1/2/4, CCND1, CDKN2D,CDC25A, MAFF, HMGA2, KCNN4, UBALD2, NEDD9, E2F1, EREG, MAPK13, NRG1, MAP3K20	DUSP8, PRUNE2, IGFBP5
SK-MEL-147MR	DUSP4/6/15,EPHA2, ETV1/4/5, SPRY2, MAFF, HMGA2, UBALD2, BCL-2, ID1, E2F1	EREG, BMP4, EpCAM, IGFBP5

Table S3. RNA sequencing in Trametinib vs. Trametinib+Nilotinib treated Resistant Cell Lines. RNA sequencing results comparing resistant lines treated with trametinib (20nM) to the same line treated with trametinib+nilotinib (2.5μM) for 24h. ERK pathway targets or genes involved in ERK pathway regulation are shown. Blue font indicates regulation in more than one resistant line. See Dataset 3 for complete gene lists.

LEGENDS FOR SUPPLEMENTARY DATASETS (Excel Files)

Dataset 1. RNA sequencing comparing parental and resistant lines. Upregulated indicates increased expression in resistant line. Downregulated indicates decreased expression in resistant line. Tab 1=SK-MEL-2; Tab 2=SK-MEL-30MR; Tab 3=SK-MEL-147MR.

Dataset 2. Whole Exome Sequencing comparing resistant lines to parental lines (removing mutations that were present in parental lines). Mutations observed in resistant lines are shown. Tab 1. WES SK-MEL-147MR vs. P—unique genes in MR. Tab 2. WES SK-MEL-147-genes in common MR and P. Tab 3. WES SK-MEL-2MR vs. P unique genes. Tab 4. WES SK-MEL-30MR vs. P unique genes. Tab 5. WES SK-MEL-30 genes in common between MR and P.

Dataset 3. mRNA changes for RNA sequencing for. Trametinib+Nilotinib vs Tametinib-Treated-Resistant Cells. RNA-seq mRNA changes between trametinib (20nM) and trametinib+nilotinib (2.5μM) treated (24h) resistant cells are shown. Tab 1. RNA-seq SK-MEL-30MR and SK-MEL-2MR trametinib vs. trametinib+nilotinib—upregulated genes in [trametinib+nilotinib] in common between 2 lines. Tab 2. RNA-seq SK-MEL-30MR and SK-MEL-2MR trametinib vs. trametinib+nilotinib-downregulated genes in [trametinib+nilotinib] common between 2 lines. Tab 3. RNA-seq SK-MEL-30MR trametinib vs. trametinib+nilotinib. Tab 4. RNA-seq SK-MEL-2MR trametinib vs trametinib+nilotinib. Tab 5. RNA-seq SK-MEL-147MR trametinib vs. trametinib+nilotinib.

SUPPLEMENTARY METHODS

Plasmids and stable expressing cell lines. Constitutively active *ABL1* and *ABL2* (PP) [25] cDNAs were removed from their vectors [pSGT-*ABL1*-PP, BamH1; [25] PK1-*ABL2*-PP, EcoR1/XhoI] [27] blunted and cloned into the Swa I site of PiggyBac cumate-inducible transposable vector (Systems Bioscience; Palo Alto, CA). Constructs were transfected using Lipofectamine 2000, selected with puromycin (1μg/ml), and clones pooled (polyclonal population). MCSV-hMYC-IRES-GFP, ETS1 (CMV-ETS1-IRES-dGFP) or beta-catenin (pEGFP-C1-beta-catenin) were transiently transfected using Lipofectamine 2000, replated into 96-well dishes, drug-treated and cell viability assessed (below).

Western Blotting. Cells were lysed in RIPA buffer (50mM HEPES pH7.0, 150 mM NaCl, 10% glycerol, 1% triton-X-100, 1.5 mM $MgCl_2$, 1mM EGTA, and fresh inhibitors (aprotinin, leupeptin, pepstatin all 10μg/ml), 1mM PMSF, 25mM NaF, 1mM sodium orthovandate. Equal amounts of cell lysate (BCA protocol; BioRad; Hercules, CA) were run on 8% SDS-PAGE gels, transferred to nitrocellulose, blocked and incubated with antibodies using the following dilutions, blocking agent, and wash conditions, respectively. ABL1 (1:1000; 0.1% tween-20); ABL2 (1:1000 5% milk, 0.05% tween); AKT (1:5000, 5%BSA, 0.1%tween); pAKT (1:1000, 5%BSA, 0.1% tween); ARAF (1:1000, 5%milk, 0.1%tween); beta-actin (1:40,000, 0.2% tween); beta-catenin (1:1000, 5%milk, 0.1%tween); BRAF (1:2000-1:10,000, 5%milk, 01.%tween), cleaved caspase (1:5000, 5%milk, 0.1%tween); cleaved PARP (1:1000, 5%BSA, 0.1%tween); CRAF (1:500, 5%milk, 0.1%tween); pCRAF (1:1000, 5%BSA, 0.1%tween); CRKL (1:2000, 5%BSA, 0.1%tween); pCRKL (1:1000, 5%BSA, 0.1%tween); cyclin D (1:1000, 5% milk, 0.1% tween); DDR1 (1:1000,

5% milk, 0.1% tween); pDDR1/2 (1:2000, 5% BSA, 0.1% tween); ERK1/2 (1:5000, 0.2% tween); pERK1/2 (1:10,000, 0.1%BSA, 0.05%tween); ETS1 (1:2000, 3%milk, 0.1%tween); pETS1 (1:1000, 5%BSA, 0.1%tween); FRA (1:1000, 5%BSA, 0.1% tween); pFRA (1:1000, 5%BSA, 0.1% tween); GAPDH (1:50,000, 0.2%tween); GFP (1:2000, 5% BSA, 0.1% tween); Histone H3 (1:1000, 5%milk, 0.1%tween); MEK1/2 (1:5000, 5%BSA, 0.1%tween); pMEK1/2 (1:2000, 5%BSA, 0.1% tween); MYC (1:1000, 5% milk); NRAS (1:5000, 5%milk, 0.1%tween); p27/KIP1 (1:1000, 5%BSA, 0.1% tween); RSK (1:500, 3%milk,0.1%tween); pRSK (1:2000, 5%BSA, 0.1%tween); anti-mouse-HRP (1:3000; 0.1%tween); LC3B (1:1000, 5% BSA, 0.1% tween; anti-rabbit-HRP (1:3000, 0.1%tween); anti-goat-HRP (1:3000, 0.1%tween). Antibody details can be found in **Table S3**; above).

***In Vitro* Kinase Assays/Mass Spectrometry.** Recombinant, full-length, kinase-inactive His-tagged, kinase-inactive forms of ABL1 and ABL2 (500ng; see **Table S3**) were incubated with full-length, GST-tagged ERK2 (20ng, see **Table S3**) or equal volume of supplied ERK2 diluent in a "cold" *in vitro* kinase assay in 20 mM Tris (pH 7.5), 20 mM NaCl, 1 mM DTT, 10 mM $MgCl_2$, 1 mM $MnCl_2$, and 1mM ATP (in HPLC-pure water) for 30 minutes at 37°C [22, 67]. Liquid chromatography-electrospray ionization-tandem mass spectrometry (LC-ESI-MS/MS) Analysis. All mass spectra reported in this study were acquired by the University of Kentucky Proteomics Core Facility. The protein gel bands were excised and subjected to dithiothreitol reduction, iodoacetamide alkylation, and in-gel trypsin digestion using a standard protocol. The resulting tryptic peptides were extracted, concentrated and subjected to shot-gun proteomics analysis as previously described [68]. LC-MS/MS analysis was performed using an LTQ-Orbitrap mass

spectrometer (Thermo Fisher Scientific, Waltham, MA) coupled with an Eksigent Nanoflex cHiPLC™ system (Eksigent, Dublin, CA) through a nano-electrospray ionization source. The peptide samples were separated with a reversed phase cHiPLC column (75 μm x 150 mm) at a flow rate of 300 nL/min. Mobile phase A was water with 0.1% (*v*/*v*) formic acid while B was acetonitrile with 0.1% (*v*/*v*) formic acid. A 50 min gradient condition was applied: initial 3% mobile phase B was increased linearly to 40% in 24 min and further to 85% and 95% for 5 min each before it was decreased to 3% and re-equilibrated. The mass analysis method consisted of one segment with 10 scan events. The first scan event was an Orbitrap MS scan (300-1800 m/z) with 60,000 resolution for parent ions followed by data dependent MS/MS for fragmentation of the 9 most intense multiple charged ions with collision induced dissociation (CID) method. MS/MS Protein Identification. The LC-MS/MS data were submitted to a local mascot server for MS/MS protein identification via Proteome Discoverer (version 1.3, Thermo Fisher Scientific, Waltham, MA) against a custom database containing only ABL and ARG (IVGN and His_KR) proteins. Typical parameters used in the MASCOT MS/MS ion search were trypsin digestion with a maximum of two miscleavages, cysteine carbamidomethylation, methionine oxidation, as well as serine, threonine, and tyrosine phosphorylation. 10 ppm precursor ion and 0.8 Da fragment ion mass tolerances. MS/MS spectra of peptide with multiple potential phosphorylation sites were manually inspected and the preferred site was selected.

Next Generation Sequencing. DNA and RNA from parental and resistant cell lines (parental vs. resistant) and resistant lines treated with trametinib vs. trametinib+nilotinib; (24h treatment) were sequenced by BGI Genomics (Shenzhen, Guangdong, China), and

data analyzed by the University of Kentucky Markey Cancer Center Biostatistics and Bioinformatics Shared Resource Facility (see below). Whole Exome Sequencing. Sequencing reads were trimmed and filtered using Trimmomatic (V0.39) [60], and aligned to the human reference genome b37/hg19 using BWA (V. 0.7.17) [69]. PCR duplicates were removed using Picard (v2.20.2; http://broadinstitute.github.io/picard/). The Genome Analysis Toolkit (GATK v4.1.2.0) [61] was used for base quality score recalibration. For comparisons between resistant lines and their parental counterparts, somatic mutations present in the resistant line but not in the parental line were detected using Mutect2 [62] tumor/normal mode with the parental line as the reference. Mutations passing the contamination filter and orientation bias filter were annotated (Oncotator, v1.9.9.0) [63]. See **Table S1** for Accession numbers.

RNA Sequencing of Cell Lines. Sequencing reads were trimmed and filtered using Trimmomatic (v0.39) [60] to remove adapters and low-quality reads. Reads were mapped to the human reference genome (GRCh38) using RSEM (v1.3.2) [64]. Differential expression analysis was performed using EdgeR (v3.32.1) [65] comparing parental vs. resistant or trametinib vs. trametinib+nilotinib samples. Significantly differentially expressed genes were determined as fold change >= 2 and q-value < 0.05. The gene set enrichment analysis was performed using GSEA (v4.0.3) [66] based on the BIOCARTA pathway database [70] KEGG pathway database [71] and REACTOME pathway database [72]. See **Table S1** for Accession numbers.

Analysis of Existing RNA sequencing Dataset. RNA-seq data obtained Dr. Yardena Samuels (Rehovot, Israel) from 23 patients (harbor NRAS-mutant melanomas) treated with MEKi (obtained from Dr. Mitchell Levesque) were utilized [46]. Spearman's

rank-order correlation analysis (R, v4.0.0) was performed to test the correlations between *ABL1* and *MYC* in MEKi-treated patient samples.

qPCR. Primer Blast (NCBI website) was used to design primers and rule out homology to other genes. RNA was isolated with RNAeasy kit (Qiagen; Germantown, MD), DNase-treated with (Ambion DNase cleanup kit), and RNA was converted to cDNA (iScript; Bio-Rad; Hercules, CA). Fifty nanograms of cDNA was amplified using SYBR green and gene-specific primers (500nM; 40 cycles, 60°C annealing temperature; CFX96, Biorad). RPS13 was used as the reference gene. Results were analyzed with CFX Manager (Bio-Rad) [73].
